# Dimension learning based chimp optimizer for energy efficient wireless sensor networks

**DOI:** 10.1038/s41598-022-18001-5

**Published:** 2022-09-02

**Authors:** Preeti, Ranjit Kaur, Damanpreet Singh

**Affiliations:** 1 Department of Electronics and Communication Engineering, Punjabi University, Patiala, India; 2 Computer Science and Engineering, Sant Longowal Institute of Engineering and Technology, Patiala, India

**Keywords:** Energy science and technology, Engineering

## Abstract

Wireless sensors are the basic requisite of today’s smart infrastructure based on internet of things (IoTs), 5G and wireless sensor networks (WSNs). WSNs are widely used in industrial applications, precision agriculture and animal tracking systems, environment monitoring, smart grids, energy control systems, smart buildings and entertainment industry etc. The distributed and dynamic scheme of WSNs establishes very unique demands in developing clustering and routing protocols. In order to meet the demand of efficient WSNs, most important requirement is energy management and extension of network lifetime. So energy constraints issue is one of the most emerging area for research to reduce the complexity of network functioning. Due to the complexity of this task we need more robustness optimizer algorithms which can tackle these types of tasks. In this article we are trying to develop one improved version of chimp optimizer for energy constraint issues. In this modification have been integrated the chimp optimizer with dimension learning based hunting (DLH) search technique, known as Improved Chimp Optimizer Algorithm (IChoA). Here the DLH search strategy helps in maintaining diversity and improves the balance between exploitation and exploration. To compute the robustness in solving the optimizer issues, IChoA has been tested on 29-CEC-2017 test suites and energy constraint issues. Experimental solutions obtained by proposed methods are verified with recent methods. All simulation shows that the IChoA method can be most effective in solving the standard complex suites and energy constraint issues.

## Introduction

Recently, WSN has increased attention among users and researchers as a powerful technological platform with tremendous applications. It has become a significant technology for understanding many applications including both simple phenomena monitoring applications and heavy duty data streaming applications such as military operations, environment monitoring and surveillance systems^1^. WSN is equipped with self-supported battery power and has limited computation power, battery life and memory resources, so maximizing the network lifetime, energy conservation measures are essential for enhancing the performance of these networks. WSN has the ability to sense, process and communicate. The most challenging concern in WSN design is how to save node energy while maintaining the desirable network performance^2^. Any WSN can only meet its mission as long as it is considered alive and hence the lifetime is an important parameter for the efficient working of WSN and can be extended by jointly applying various energy efficient techniques. Clustering is one of the approaches to enhance the network lifetime by introducing energy balancing and data dissemination amongst the sensor nodes (SNs)^3^. For clustered networks, data collected by each node within the cluster is forwarded to cluster head (CH) and then the data aggregated by CH is transmitted to base station (BS) over the long distances and most of the energy is drained during this process by cluster heads (CHs) as compared to other sensors. Its mandatory to avoid this fast energy depletion of CH. The optimal choice of CHs, their reconfiguration in different rounds and maintaining cluster are the main issues to be considered during development of the clustering algorithms^4^.

Another challenge is optimization of the energy consumption which can make the wireless communication system perform better. So, conservation and optimization of energy is the recent research area still need to explore for better performance evaluation of WSNs. There are many existing algorithms or protocols aiming at improving the performance of sensor networks, such as Low-Energy Adaptive Clustering Hierarchy (LEACH)^5^, Mean Shift Clustering Algorithm (MEAN-SHIFT)^6^, Threshold-Sensitive Energy Efficient Sensor Network (TEEN)^7^, a Hybrid Energy-Efficient Distributed Clustering Approach (HEED)^8^, K-Mean Clustering Algorithm (K-MEAN)^9^, Linked Clustering Algorithm (LCA)^10^, Energy-Efficient Clustering Scheme (EECS)^11^, Hierarchical Clustering Algorithm (HC)^12^, High Energy First Algorithm (HEF)^13^, Energy Residue Aware (ERA)^14^, Fuzzy C Mean Clustering Algorithm (FCM)^15^, and many more. All methods above provide the improvement in power utilization in communication and lengthen the network lifetime in one way or the other depending upon the scenario of WSN. Also, clustering enhances the stability and ensures the reliability of the network. these are traditional approaches for WSN and further can be enhanced by using optimization algorithms.

The use of Optimization algorithm is to obtain the optimum or best result under a given circumstances. The word optimum is taken as maximum or minimum depending on circumstances. The sensor network protocols have to focus primarily on saving the power for lengthening network life and other issues are, attaining high quality QoS, low bandwidth, processing speed and limited storage in each node^16^ and the solution is directly associated to the optimization. This is not possible to replace batteries in remote areas due to hostile geographical locations.So, battery life in sensor node is a crucial concern to be addressed. With a very little infrastructure for WSN, the deployment of the sensor nodes is random and the base station is either inside the monitoring area or very near to it^17^. There are several optimization algorithms to suit the different problems and choosing a proper algorithm is very important.

Most of the WSN problems have many difficulties consisting of large space solutions, non linear limits, non-convex search landscape, energetic / strident objective functions and maximum computational cost. These can be solved by utilizing the optimal solutions for the variable result to minimize or maximize an objective function irrespective of varying limit values^18^. While the proper and precise solution may be provided by the accurate algorithm for calculating global optimum value and time for execution of numerous variables is increased exponentially. On the other hand stochastic techniques are capable of finding the nearest optimal solution within stimulated period of time. They can be classified as heuristic and metaheuristic algorithms. The metaheuristic algorithms can be further divided into two groups such as nature inspired algorithms and non nature inspired algorithms. Numerous metaheuristic algorithms are nature inspired such as Particle Swarm optimization (PSO), Artificial Bee Colony (ABC), Genetic Algorithm (GA), Ant colony optimization (ACO) and many more where as a few are non nature inspired such as tabu search, adaptive dimension search and iterative local search etc.^19^.

Some more nature inspired optimization algorithms incorporating evolutionary model and swarm Intelligence are used for WSN are Firefly Algorithm (FA), BEES optimization Algorithm (BOA), Particle Swarm Frog leaping hybrid optimization algorithm, Elephant swarm optimization, Cuckoo search (CS), and Bat Algorithm (BA)^20^.

This paper introduces an improved version that integrates the phases of Chimp Optimizer and dimension learning based hunting (DLH) search strategy. In this modification the DLH phase plays a role for maintaining diversity and also improves the balance between exploitation and exploration. Numerical and statistical experiments have been done for comparing the performance of the metaheuristics. To sum up, the major contributions of this research work are:

- An improved algorithm namely IChoA that includes features from ChoA and DLH search strategy is proposed.
- IChoA is presented for solving 29-CEC-2017 test functions.
- The proposed algorithm is presented for deal with energy constraint issues.
- IChoA based clustering save energy spending the density criterion and residual energy of nodes.
- IChoA based routing achieves data sending using intra-cluster multipath communications.
- Statistical and qualitative experimental analyses assess the effectiveness of the ICHOA method compared to recent algorithms.

The rest of the paper is organized as follows: In “Literature review”, a literature review is presented. In “Chimp optimization algorithm (ChoA)”, the various material regarding the basic of chimp optimizer is introduced. The “The proposed IChoA algorithm” explains the improved IChoA method. The “Results and discussion” results discussion and analysis the obtained numerical and statistical solutions. In “Formulation of energy constraints problem”, the energy constraint functions have been formulated.The working process of IChoA method on energy constraint issues is reported in “Implementation”. In “Simulation outputs and discussion” results discussion and analysis the obtained numerical and statistical solutions of the energy constraint issues. Finally, “Conclusion” provides conclusions for this paper.

## Literature review

The life span of the battery of each sensor device is limited which severely constrains the operation of the sensor network. Therefore, proper schemes must be proposed to tackle this problem so that the exploitation of sensor networks can become practical. The key point to develop a clustering algorithm is distribution strategy of nodes, cluster formation method, data dissemination process, communication protection, stability, synchronization etc. The exhaustive literature is reviewed in this section as follows:

Singh et al.^21^ designed concentric layered architecture (CLA) for optimal location of layers and numbers of SN in every layer in static and random WSN multi level clustering. CLA divided the entire area into layers depending upon nodes in every layer and node density. CLA divided the WSN into concentric layers for numerous SNs so that the outer later consisted of two times compared to internal layers to avoid intra cluster load. CLA evaluated for PSO-C and simulation results showed that it performed appreciably in terms of energy efficiency and prolonged lifetime compared to PSO-ECHS, BERA, and PSO-C.

Purkar et al.^22^ invented an energy efficient clustering Protocol to enhance for performance of heterogeneous WSN (EECPEP-HWSN). The proposed protocol divided the nodes into three different levels known as normal, advance and super nodes. The Cluster formed and CH elected on the basis of starting energy, hop count and remaining residue power. The EECPEP-HWSN protocol outperformed in comparison to existing protocols 188% better than LEACH, 150% than DEEC and 141% than SEP. Also, contributed to improve network lifetime, stability, energy efficiency and remaining energy in the network.

Singh et al.^23^ proposed multilevel clustering protocol (MLCP) for load balancing and energy efficient data aggregation in large scale scalable WSNs. Hierarchal clustering architecture developed for clustering and routing issues. CH selection done using hybrid algorithms of dragonfly and PSO. MLCP considered various parameters of network such as node degree, inter and intra cluster distance for scalability, load balancing and energy efficiency. The simulated results for MLCP proved the increase of 90% in lifetime and 19.36% in energy consumption in comparison to existing techniques BERA, PSO-ECHS and PSO-C for achieving load balancing and energy efficiency.

Inam et al.^24^ introduced energy efficient clustering and shortest path routing Protocol (EECSRP) for WSNs by reducing network overhead, enhancing network lifetime, maximum PDR and energy conservation. The CH selected on the basis of residue energy, node degree and RSS (received signal strength). Also, RSS based network distribution implemented for estimating gradient based on demand path selection between SN and BS. During the clustering process, when CH residual energy attained the threshold level and reduced the transmission for control packets. To avoid the data collision and MAC layer conflict BS collected data from every CH. The simulated results provided consistent performance of the network in terms of average energy consumption, pack delivery ratio, controlled overhead and average end to end delivery compared to the existing protocols i.e. R-LEACH, CCMAR and EESAA.

Mood et al.^25^ modified the GSA (Gravitation search algorithm) for energy efficient clustering in WSNs. The proposed algorithm efficiently organized these clusters and calculated optimum number of CH depending upon the link quality and energy consumption. A new fitness function is proposed to organize the compact clusters by modifying GSA for calculating the mass values of power distance sums scaling technique. The fuzzy controller employed for identifying performance metrics of this technique for exploitation and exploration. The method presented minimized number of active CH to reduce energy conservation. The simulated results discovered that modified GSA performed better than existing metahueristics such as LEACH PSO-C, CSA, GSA and PDSS-GSA to enhance the network lifetime for energy efficient WSNs.

Raju Pal et al.^26^ developed a new GA based energy efficient clustering weighted clustering (EEWC) for heterogeneous WSN. New weighted fitness objective function provided the modified steady state phase of LEACH for three important clustering constraints named as density, partition and numeral cluster heads. The proposed optimization function used to find the finest sets for CH in SS phase of LEACH by virtue of GA. The simulated outcomes improved the performance of WSN as compared to SEP, ERP and IHCR in terms of network lifetime, stability and complete residue energy.

Delgado et al.^27^ presented a DCAGBS approach (distributed clustering algorithm guided by base station) to improve the network life time of the WSNs. The clusters formed dynamically with the help of BS and here BS sent three information data for the time to reconfigure SKIP value (number of round where CHs have same values) in accordance with fuzzy logic approach named as TAGAKI-Sugeno-Kang model. The concluded results showed that the centralized techniques are not feasible as they relayed too much information on severs that in turn increased the size and cost of the network so proposed distributed fuzzy based system for section of CH with dynamically configured BS. The proposed DCAFBS algorithm compared with the existing distributed and centralized methods (LEACH, CHEF, EEDCF, and EUDFC) to evaluate the network lifetime.

Singh et al.^28^ proposed a EESCP (energy efficient scalable clustering protocol) for balanced clustering based on inter an intra cluster distance to form clusters of equal size among the entire network. PSO based Dragon fly algorithm is employed for selecting cluster heads and cost of communication is reduced by minimizing the intra cluster distance to achieve the maximum network coverage. The simulated results implemented for various network densities and sizes and EESCP outperformed in comparison to other algorithms such as BBO, DE, ABC, PSO in regards to balanced cluster formation, rotation of cluster head, network lifetime, energy consumption and throughput. Also, the WSN function performed robustly for different network densities and sizes.

Sharma et al.^29^ presented eeTMFO/GA (energy efficiency trusted moth flame optimization and genetic algorithm) for CH selection and the fitness function evaluated for residue energy, average cluster distance, node density, packet transmission and delay in transmission. The proposed optimizer MFO for CH election provided the overall enhancement of reliability and security of the network. The implemented results performed better for stable network and energy consumption compared to existing protocols such as 56.09% from HEED, 60% from LEACH and proved the better performance 42.26% and 16.36% with ABC and QABC respectively. Also, eeTMFO/GA showed huge reduction in electing malicious sensor nodes to be selected as CH as compared to ALM, TCM.

Qin et al.^30^ proposed a distributed clustering algorithm with normalized information measures for sensor networks. Initially, normalized information distance (NID) selected for cluster data and label by minimizing the objective function. And then by introducing minimum normalized information distance based algorithm (MNID) by minimizing NID for model selection by exploring the capabilities of non Gaussian data clustering. The MNID algorithm implemented for distributing clustering employed for finite time multi agent consensus to calculate the global parameters where local variable exchanged for single hop neighbors. MNID algorithms simulated for both centralized and distributed network coverage by numerically tested for both synthetic and real data. The proposed algorithm outperformed in comparison to existing algorithms in terms of network performance.

Ullah et al.^31^ investigated clustering protocols for network lifetime and SNs energy based on HEED for WSNs and summarized the advantages and disadvantages. The focus aimed to enhance network lifetime and energy consumption parameters for quality of service for both homogeneous and heterogeneous WSN. The analysis provided helpful information based on various HEED algorithms such as DWEHC, RUHEED, HEED-NPF, HELPER-HEED, U-HEED, H-HEED, D-HEED AND fuzzy based H-HEED.

Nadimi-Shahraki et al.^32^ proposed an improved grey wolf optimizer (I-GWO) for solving the engineering design optimization problems by developing premature convergence, lack of population diversity and imbalance between exploitation and exploration capability of GWO algorithm. I-GWO provided DLH (dimension learning based hunting) approach inherited from independent hunting of wolves in nature which provided balance in-between global and local search for maintaining diversity. The results for I-GWO simulated for uni-modal, multi-modal, hybrid and composite bench mark functions and are compared to various existing techniques such as PSO, KH, GWO, WOA,EEGWO, HGSO and proved that proposed I-GWO outperformed for various statistical tests and experiments for well suited for engineering problems and power flow issues.

Devassy et al.^33^ proposed a novel bio-inspired algorithm (NBA) protocol by combining LEACH with dragonfly algorithm for CH selection. The dragonfly algorithm utilized for deploying sensor nodes and selecting best possible route for delivery of packets to BS. The proposed algorithm performed better in expanding network lifetime as compared to existing algorithms such as LEACH, MS-LEACH, SFFA and ESO-LEACH) in terms of dead nodes, PDR and number of live nodes.

Bangotra et al.^34^ proposed two nature inspired routing algorithms for attaining energy efficiency and secure WSN named as intelligent opportunistic routing protocol (IOP) and trust based secured secure intelligent opportunistic routing protocol (TBSIOP). The proposed algorithms compared with ACO and PSO terms of energy efficiency, average risk level, network lifetime, end to end delivery and packet delivery ratio.

## Chimp optimization algorithm (ChoA)

Freshly, chimp optimizer is a newly population based nature inspired algorithm is developed by Khishe et al.^35^ for handling the complex real issues. This metaheuristic method is motivated by sexual inspiration and specific intelligence of chimps which is famous for their own group hunting. The robustness of this method shows yourself different from others. During finding the best target it works under four categories as barrier, driver, attacker and chaser respectively. The details of these categories are illustrated under the following steps as;

Firstly, chasing and driving phase is amended by the following Eqs. (1, 2)

1 $$\begin{aligned}&D=\left| c.a_{prey}\left( n \right) -ma_{chimp}\left( n \right) \right| \end{aligned}$$

2 $$\begin{aligned}&a_{chimp}\left( n+1 \right) =a_{prey}-a.d \end{aligned}$$

where *n*, *c*,*m* and *a* is denoted the number of iterations and coefficient vectors. The coefficient vectors are amended by the Eqs. (3, 4);

3 $$\begin{aligned} a= & {} 2.l.r_1-l \end{aligned}$$

4 $$\begin{aligned} c= & {} 2.r_2 \end{aligned}$$

5 $$\begin{aligned} m= & {} chotic_{value} \end{aligned}$$

where $r_1$ and $r_2$ are illustrates the random constants and these lies amid $\left[ 0,1 \right]$. Further *m* illustrates the chaotic vector and *l* is reduces non-linearly from 2.5 to 0 through the generation process.

The behavior of each chimp is amended by mathematically under this stage. It assumed the first solution is available by the driver, attacker, chaser and barrier are superior information conveyed about the position of the best target. First four best solutions are stored for updating the next new position and rest members are forced to update their own location according to the best search agents location in the search space during the search process. This process is done in the search space by the following Eqs. (6)–(9).

6 $$\begin{aligned} d_{attacker}= & {} \left| c_1a_{attacker}-m_1.x \right| \end{aligned}$$

7 $$\begin{aligned} d_{barrier}= & {} \left| c_2a_{barrier}-m_2.x \right| \end{aligned}$$

8 $$\begin{aligned} d_{chaser}= & {} \left| c_3a_{chaser}-m_3.x \right| \end{aligned}$$

9 $$\begin{aligned} d_{driver}= & {} \left| c_4a_{driver}-m_4.x \right| \end{aligned}$$

However, when the value of the random parameter range falls between $\left[ -1,1 \right]$, then the next position of search agent could be in any position between the current and prey location.

10 $$\begin{aligned} x_1= & {} a_{attacker}-a_1.d_{attacker} \end{aligned}$$

11 $$\begin{aligned} x_2= & {} a_{barrier}-a_2.d_{barrier} \end{aligned}$$

12 $$\begin{aligned} x_3= & {} a_{chaser}-a_3.d_{chaser} \end{aligned}$$

13 $$\begin{aligned} x_4= & {} a_{driver}-a_4.d_{driver} \end{aligned}$$

With the help of previous equations, the position of each member is evaluate by the following Eq. (14);

14 $$\begin{aligned} x_{n+1}=\frac{x_1+x_2+x_3+x_4}{4} \end{aligned}$$

Lastly, the following Eq. (15) has been used for evaluating and modifying the position of each search member during the search process;

15 $$\begin{aligned} a_{chimp}\left( n+1 \right) =\left\{ \begin{array}{lll} a_{prey}\left( n \right) -x.d,&{} if &{} \phi <0.5 \\ chaotic_{value}&{} if &{} \phi >0.5 \end{array}\right. \end{aligned}$$

### Pseudocode of ChoA

The pseudocode of CHoA algorithm is reported in Algorithm 1.
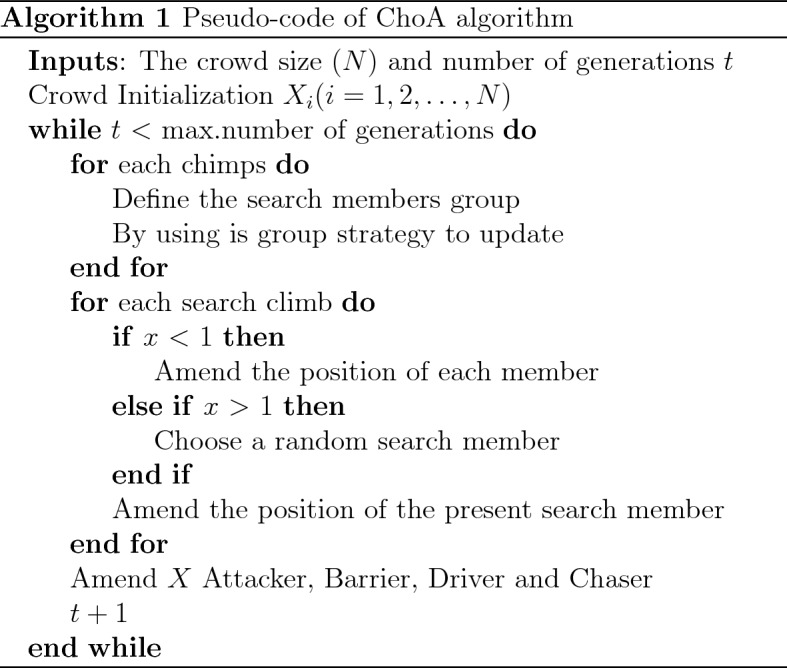


## The proposed IChoA algorithm

The various complex domain real world optimization tasks related to energy constraint issues in wireless sensor networks are huge challenges for the optimizer methods. Last few decades, a large number of robust optimizer methods have been developed by scientists. But due to their various shortcomings these are not able to prove their own robustness for complex domain issues. Each optimizer method may be faced with some shortcomings for finding the best solution in the complex domains, so being that reasons these methods could be failure to trap the best optima location in the search domain for these complex suites.

Hence, due to the current demand of the various domains of engineering, computer science and many more, we need a huge amount of most powerful optimizer methods which would fulfill the current and future demands related to Wireless Sensor Networks (WSN), engineering applications and many other optimization tasks. As an example, these methods could easily tackle complex tasks, if the exploration and exploitation phases of these optimizer methods would be most powerful.

In this work, we are trying to introduce a newly enhanced version of chimp optimizer for tackling the energy constraint issues in wireless sensor networks. This version also helps to overcome the various shortcomings of the chimp optimizer such as imbalance amid the exploitation and exploration, diversity and premature convergence respectively. The proposed method benefits from a new drive technique known as dimension learning based hunting (DLH) search technique congenital from the specific chasing performance of the search members in search space. This strategy applies to construct a local region for every search member in the search space in which the best region information can be shared amid search members. Under this methodology, the DLH strategy helps in maintaining diversity and improves the balance between exploitation and exploration.The implementation stages of the proposed method are illustrated in detail by the following steps;

### Constant settings

Over the implementation of the code of the optimizers have been taken various settings of the parameters such as size of crowd (*n*=30), constants ($c_{3}=1$ $c_{4}=2$), size of generations (500), lb (-100) and ub (100) respectively.

### Initialization

The initialization procedure is usually the same taken in each nature inspired algorithm, in the search domain the position of each member signifies the outcome for the decision variables. The following mathematical Eqs. (16)–(19) are used to evaluate the places of all members;

16 $$\begin{aligned} M= \begin{bmatrix} m_{1,1} &{} m_{1,2} &{}, \cdots , &{} m_{1,d}\\ m_{2,1} &{} m_{2,2} &{}, \cdots , &{} m_{2,d}\\ \vdots &{} \vdots &{} \ddots &{} \vdots \\ m_{n,1} &{} m_{n,2} &{}, \cdots , &{} m_{n,d}\\ \end{bmatrix} \end{aligned}$$

where *n* and *d* signifies the size of crowd and size of function.

For fitness of population has been utilized the following Eq. (17);

17 $$\begin{aligned} FM=\begin{bmatrix} fm_{1} \\ fm_{2} \\ \vdots \\ fm_{n} \\ \end{bmatrix} \end{aligned}$$

where $fm_{i}$ are denotes the fitness value of the *i*th member. In the same way, the subsequent matrices can be express for the target by mathematical Eqs. (18) and (19);

18 $$\begin{aligned} P= & {} \begin{bmatrix} p_{1,1} &{} p_{1,2} &{}, \ldots , &{} p_{1,d}\\ p_{2,1} &{} p_{2,2} &{}, \ldots , &{} p_{2,d}\\ \vdots &{} \vdots &{} \ddots &{} \vdots \\ p_{n,1} &{} p_{n,2} &{}, \ldots , &{} p_{n,d}\\ \end{bmatrix} \end{aligned}$$

19 $$\begin{aligned} FP= & {} \begin{bmatrix} fp_{1} \\ fp_{2} \\ \vdots \\ fp_{n} \\ \end{bmatrix} \end{aligned}$$

where *n*, *d* and $fp_{i}$ are illustrates the total search members, dimension and fitness value of the *i*th member.

### Fitness evaluation

The following Eqs. (20) and (21) are utilized to evaluate the fitness best and worst score of crowd ;

20 $$\begin{aligned} F_{best}= & {} Min (fit_{j}(z)) \, \, j \varepsilon (1,2,\ldots ,n) \end{aligned}$$

21 $$\begin{aligned} F_{worst}= & {} Max (fit_{j}(z)) \, \, j \varepsilon (1,2,\ldots ,n) \end{aligned}$$

### Drive stage

DLH strategy helps to specifically chase the goal of the population members in the search area. This strategy applies to construct a local region for all chimps in the search area in which the best region or neighbors information can be shared amid chimps. Further the following subsection signifies how DLH and canonical ChoA portions create different members.

#### Canonical ChoA phase

In the basic algorithm the first four finest population members are dignified as attacker, barrier, chaser and driver etc via Eqs. (6)–(9). The *a* and *c* coefficient vectors are estimated via Eqs. (3) and (4). After that, the finest place of $x_{1}$, $x_{2}$, $x_{3}$ and $x_{4}$ search members are evaluated via Eqs. (10)–(13). Finally the first crowd member for the novel place of chimp $x_{i} (n)$ named $x_{i-ChoA} (n+1)$ is amended via Eq. (14).

#### DLH phase

In basic ChoA method the place is amended via first four member of the crowd. With this fact the original algorithm offers the crowd fails diversity too early, slow convergence, and population members can stuck in the native optima. To minimizing these drawbacks, in IChoA the DLH part supports in the distinct chasing of population agents that is learned by its neighbors.

In DLH strategy, all aspect of the fresh place is amended via a mathematical equation in which this diverse population agent is learned via its dissimilar neighbors and a randomly chosen member from the population. Then, further $x_{i-ChoA} (n+1)$, the DLH phase produces supplementary population candidate for the fresh place of chimp $X_{i}(n)$ named $X_{i-DLH}(n+1)$. To do this, a radius $R_{t}(n)$ is assessed put on Euclidean distance amid existing place of population member $x_{i}(n)$ and the candidate location $X_{i-ChoA}$ via mathematical Eq. (22).

22 $$\begin{aligned} R_{i}(n)=\left\| x_{i}(n)-x_{i-ChoA}(n+1) \right\| \end{aligned}$$

Then, the near search members of $x_{i}(n)$ indicated by $L_{t}(n)$ are evaluated by Eq. (23) with respect to Eq. (22).Here $M_{i}$ shows the Euclidean distance between $x_{i}$ and $x_{i}$.

23 $$\begin{aligned} L_{i}(n)=\left\{ x_{i}(n),M_{i}( x_{i}(n),x_{j}(n))\le R_{i}(n),x_{j}(n)\epsilon \,crowd\right\} \end{aligned}$$

After that, the multi-neighbors learning is executed by the following mathematical formulation of Eq. (24);

24 $$\begin{aligned} x_{i-DLH,d}(n+1)=x_{i,d}(n)+rand \times (x_{m,d}(n)-x_{r,d}(n)) \end{aligned}$$

where *d* is illustrate the dimension.

### Position update stage

Over this part the finest population agent is designated via comparing the fitness outcome of dissimilar crowd members $x_{i-ChoA}(n+1)$ and $x_{i-DLH}(n+1)$ by equation;

25 $$\begin{aligned} x_{i}(n+1)=\left\{ \begin{array}{ll} x_{i-ChoA}(n+1) &{} if \, \, f(x_{i-ChoA})< f(x_{i-DLH})\\ x_{i-DLH}(n+1) &{} otherwise \end{array} \right. \end{aligned}$$

So, according to the previous mathematical equation if the fitness output of the chosen population agent is $< x_{i}(n)$, then $x_{i}(n)$ is amended via the selected crowd agent. Else, $x_{i}(n)$ remains unchanged in the population.

### Stopping condition

Last of all, the bring to an end conditions is taken for amending the fresh place of the population member in the search domain. This process is continuously repetitive, up until it does not satisfy the standards of prevention.

### Pseudocode of IChoA

The pseudocode of Improved Chimp Optimizer is reported in Algorithm 2.
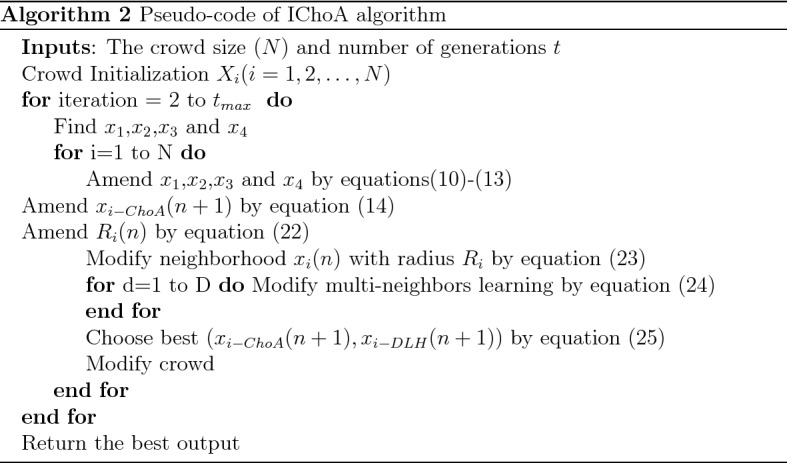


## Results and discussion

For evaluating the robustness of the proposed method has been verified by various recent meta-heuristics such as SCA^36^, Chimp^35^, SBPSO^37^ and AOA^38^ respectively. Further, the performance of the presented method has been discussed by following subsections;

### Constant and CEC’2017 test suites

The algorithms are coded in the RMatlab-2018a software and these runned on Core i3 8th Gen system, 8 GB Ram with 64 for bit operating system for comparing the robustness and effectiveness of these. During this evaluating procedure has been fixed the values of crowd (30), total iter (500), dimension of functions (10–100), lb and ub taken between − 100 to 100 etc.

**Figure 1:**
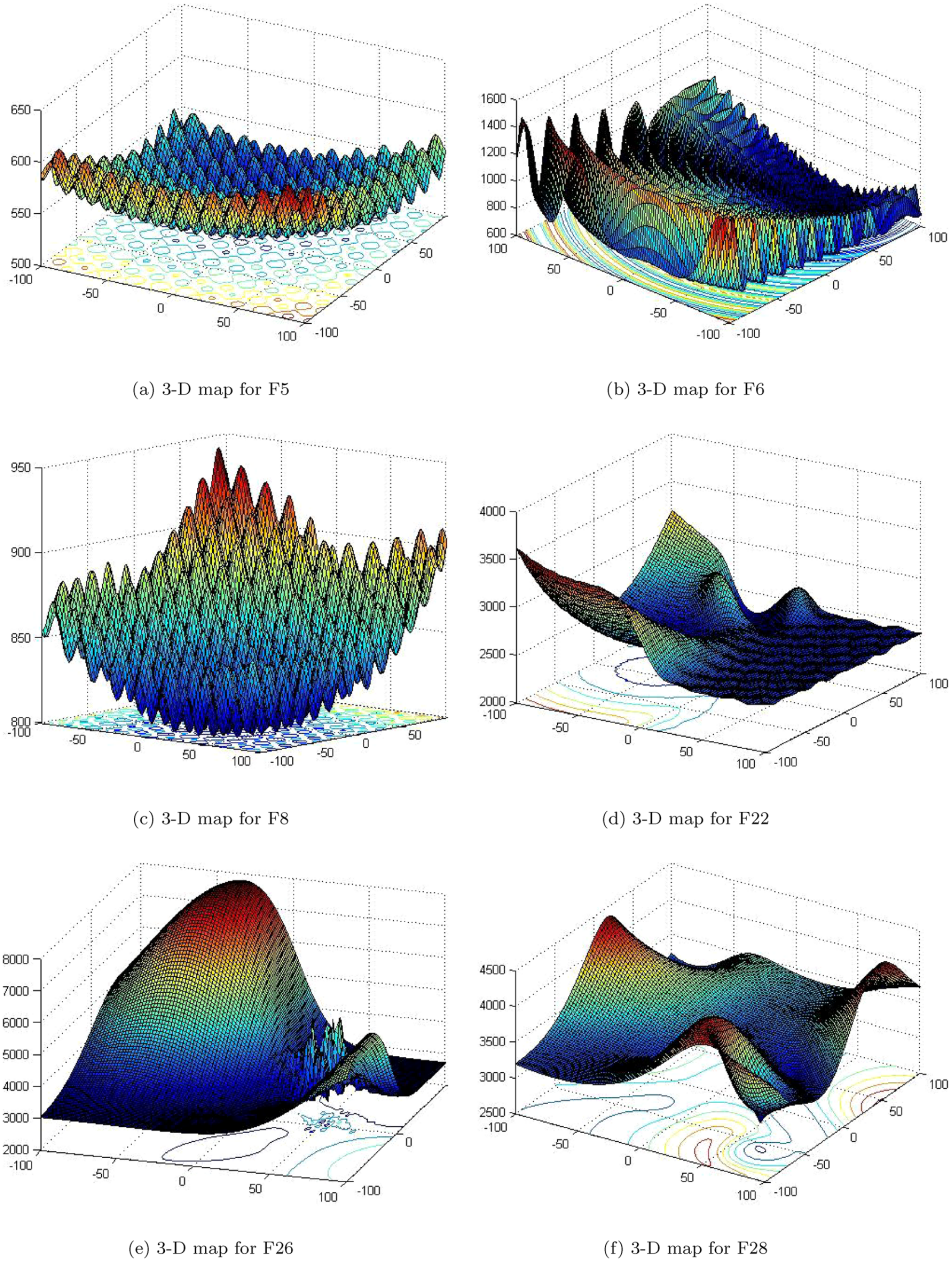
3-D graphs of CEC’2017 test suites.

For examine the robustness and efficiency of the IChoA method, a well-known standard CEC’2017 test suites have been applied and reported in Table  1^39^. The 3-D graphs of these suites are illustrated by Fig. 1. In which these suites are involves four types of functions such as Unimodal (1–2), Simple multi-modal (3–9), Hybrid (10–19) and Composition (20–29) respectively.

**Table 1:** Summary of the CEC’2017 test suite.

Name	No.	Function	$F^*_i=F(x^*)$
Unimodal	1	Shifted and rotated Bent Cigar function	100
Unimodal	2	Shifted and rotated Zakharov function	200
Simple Multimodal	3	Shifted and rotated Rosenbrock’s function	300
Simple Multimodal	4	Shifted and rotated Rastrigin’s function	400
Simple Multimodal	5	Shifted and rotated Expanded Scaffer’s F6 function	500
Simple Multimodal	6	Shifted and rotated Lunacek Bi-Rastrigin function	600
Simple Multimodal	7	Shifted and rotated Non-Continuous Rastrigin’s function	700
Simple Multimodal	8	Shifted and rotated Levy function	800
Simple Multimodal	9	Shifted and rotated Schwefel’s function	900
Hybrid	10	Hybrid function 1 (N = 3)	1000
Hybrid	11	Hybrid function 2 (N = 3)	1100
Hybrid	12	Hybrid function 3 (N = 3)	1200
Hybrid	13	Hybrid function 4 (N = 4)	1300
Hybrid	14	Hybrid function 5 (N = 4)	1400
Hybrid	15	Hybrid function 6 (N = 4)	1500
Hybrid	16	Hybrid function 6 (N = 5)	1600
Hybrid	17	Hybrid function 6 (N = 5)	1700
Hybrid	18	Hybrid function 6 (N = 5)	1800
Hybrid	19	Hybrid function 6 (N = 6)	1900
Composition	20	Composition function 1 (N = 3)	2000
Composition	21	Composition function 2 (N = 3)	2100
Composition	22	Composition function 3 (N = 4)	2200
Composition	23	Composition function 4 (N = 4)	2300
Composition	24	Composition function 5 (N = 5)	2400
Composition	25	Composition function 6 (N = 5)	2500
Composition	26	Composition function 7 (N = 6)	2600
Composition	27	Composition function 8 (N = 6)	2700
Composition	28	Composition function 9 (N = 3)	2800
Composition	29	Composition function 10 (N = 3)	2900
–	–	Search range $[-100,100]^D$	–

### Experiment and comparison

The trial must be done multiple n-times to create constant or stable statistical outputs for validating the accuracy and robustness of the metaheuristics. In this work, the same procedure has been applied for validating the performance of the algorithms. Output of the algorithms are verified in the terms of least and highest objective value, mean and standard deviation respectively. Experimental outputs and convergence performance of the metaheuristics are illustrated by Tables 2, 3 and 4 and Figs. 3, 4, 5 and 6.

Experimental results and convergence graphs show that the IChoA method is giving the effective and highly accurate solutions on 29-CEC’2017 standard test suites as comparison than others. In addition, the results have been discussed in full details in the following subsections.

#### Discussion on solutions of Tables 2, 3 and 4

This subsection is split into different categories as firstly, the uni-modal suites outputs denote the exploitation ability of the algorithms. Secondly, the various local optima are involved in multimodal suites which examine the ability of algorithms for many optima in the search space. Thirdly, the hybrid and composite test suites have been utilized to assess exploration ability of the algorithms.

#### Assessment of exploitation capability

Uni-modal test suites which involve only single global and these have been utilized for testing the exploitation phase. Experiments showed that the IChoA method has demonstrated superior exploitation ability as comparison than other recent metaheuristics. Results of Tables 2, 3 and 4, can be given strong evidence that the proposed technique is able to give the best and accurate solutions for all unimodal suites than others. Simulated results prove that this modification and enhancement of ChoA method allowed the test suites to reach the optimum score. So here it can be concluded that the IChoA method can be found with the best optima score during the search process in the search space with strong exploitation behavior which could be implemented for complex issues that need to be addressed.

As stated formerly, these suites are more compatible for evolutionary and benchmarking techniques. Experiments illustrate that the IChoA method is extremely functional. All simulations have proven that the enhancement of the ChoA method is more effective and successful in finding the best global optima for the single objective suites.

#### Capability assessment

Multi-modal test suites which involve various local optima in which the number of decision variables rises exponentially against the dimension of the test suite as compared to the uni-modal test suite. Normally these are used for verifying the suitability of the standard test suite techniques. Experiments show that the IChoA method proves the sophisticated and strong detection behavior.

#### Balance between exploration and exploitation

Hybrid and Composite test suites which involve large numbers of global and local optima. Generally these suites are used for verifying the accuracy of the algorithms in local and global optima survivors and balance amid exploitation and exploration respectively. Experiments give strong evidence that the IChoA is able to create a strong balance between exploitation and detection. With this fact, we can say that during finding the best optima score in the search area which showed the strong exploration and exploitation behavior of the proposed method. This strategy helps to create a new best location for all chimps in the search area at each generation and chimps amend their own current locations to the best location or with superior fitness in many points of view that make it possible for IChOA algorithm to reach the best score. Numerical and Statistical measurements show the strong detection behavior of the IChoA method in finding the best global values in the complex space. Because in hybrid and composite test suites involves more complex space so highly exploration and exploitation behavior based methods are needed for finding the accurate and best solution for these kinds of issues. Experiments give strong proof that the IChoA leads to best global optima with the help of their best scan behavior.

In addition, IChoA method involve a strong local optima avodance from which helps in finding best optima fastly.

**Table 2:** The best optima solutions of algorithms on the 29-CEC’2017 standard test suites.

F	SCA		Chimp		SBPO		AOA		IChoA	
f1–29	$F_{min}$	$F_{max}$	$F_{min}$	$F_{max}$	$F_{min}$	$F_{max}$	$F_{min}$	$F_{max}$	$F_{min}$	$F_{max}$
f1	6.33E+10	2.76E+11	5.63E+10	3.15E+11	1.04E+11	1.38E+11	5.44E+10	3.12E+11	4.90E+09	3.55E+11
f2	3.14E+03	2.71E+06	3.04E+03	1.22E+05	3.47E+04	3.49E+04	3.90E+03	3.63E+05	4.07E+02	8.77E+06
f3	5.19E+04	3.43E+05	4.34E+04	2.96E+05	5.23E+04	1.70E+05	3.55E+04	2.92E+05	1.13E+04	2.06E+05
f4	2.06E+03	3.26E+03	2.03E+03	3.10E+03	2.27E+03	2.78E+03	1.98E+03	3.28E+03	1.76E+03	3.49E+03
f5	7.04E+02	7.74E+02	6.96E+02	7.66E+02	7.07E+02	7.36E+02	6.96E+02	7.72E+02	6.76E+02	7.79E+02
f6	4.20E+03	1.28E+04	3.68E+03	1.35E+04	1.08E+04	1.27E+04	3.74E+03	1.26E+04	3.10E+03	1.42E+04
f7	2.36E+03	3.59E+03	2.34E+03	3.62E+03	2.45E+03	2.55E+03	2.33E+03	3.66E+03	2.06E+03	3.84E+03
f8	8.49E+04	2.16E+05	7.50E+04	2.66E+05	1.19E+05	1.39E+05	7.67E+04	2.32E+05	6.03E+04	2.76E+05
f9	2.42E+03	3.35E+03	3.09E+03	3.94E+03	3.16E+03	3.27E+03	2.63E+03	3.87E+03	2.31E+03	3.97E+03
f10	1.87E+05	3.28E+07	2.30E+05	5.35E+08	2.09E+05	2.51E+05	1.54E+05	9.51E+06	8.77E+04	5.56E+07
f11	9.60E+10	4.39E+11	1.01E+11	3.88E+11	1.66E+11	2.61E+11	8.14E+10	3.91E+11	1.83E+10	5.91E+11
f12	2.18E+10	1.12E+11	2.66E+10	1.07E+11	3.28E+10	5.79E+10	1.22E+10	1.10E+11	2.16E+09	3.04E+11
f13	9.63E+07	7.19E+08	2.50E+07	6.39E+08	8.46E+07	1.72E+08	4.05E+07	3.93E+08	6.96E+06	9.72E+08
f14	5.90E+09	6.74E+10	1.05E+10	4.55E+10	1.76E+10	3.16E+10	5.43E+09	4.71E+10	1.09E+08	6.80E+10
f15	1.55E+04	5.17E+04	1.53E+04	5.62E+04	1.77E+04	2.18E+04	1.48E+04	3.16E+04	1.04E+04	5.70E+04
f16	1.02E+05	3.90E+08	1.23E+04	7.16E+07	4.34E+06	7.28E+06	2.88E+05	2.58E+08	7.17E+03	4.11E+08
f17	3.79E+07	2.32E+09	4.45E+07	1.86E+09	8.93E+07	2.12E+08	9.50E+07	1.57E+09	1.72E+07	2.89E+09
f18	8.26E+09	3.99E+10	1.22E+10	3.90E+10	1.97E+10	3.34E+10	6.68E+09	6.26E+10	8.79E+07	6.83E+10
f19	8.31E+03	9.96E+03	7.98E+03	9.97E+03	7.98E+03	8.44E+03	8.27E+03	9.70E+03	7.82E+03	1.01E+04
f20	4.26E+03	5.65E+03	4.35E+03	5.22E+03	4.20E+03	4.66E+03	3.98E+03	5.65E+03	3.50E+03	5.73E+03
f21	2.43E+03	3.71E+03	4.08E+03	4.97E+03	3.57E+03	3.57E+03	2.84E+03	4.69E+03	2.30E+03	5.75E+03
f22	5.38E+03	7.54E+03	5.43E+03	8.14E+03	4.28E+03	4.73E+03	6.07E+03	8.42E+03	4.02E+03	9.30E+03
f23	3.92E+03	5.63E+03	4.00E+03	5.46E+03	3.67E+03	4.04E+03	4.28E+03	6.06E+03	3.34E+03	6.98E+03
f24	9.21E+03	6.35E+04	1.22E+04	7.86E+04	2.71E+04	3.55E+04	8.64E+03	5.29E+04	3.55E+03	7.95E+04
f25	1.49E+04	2.53E+04	1.44E+04	3.49E+04	1.18E+04	1.61E+04	1.37E+04	3.17E+04	8.40E+03	3.25E+04
f26	4.87E+03	6.79E+03	4.89E+03	7.76E+03	3.93E+03	4.60E+03	4.58E+03	1.01E+04	3.76E+03	2.06E+04
f27	8.77E+03	2.65E+04	8.52E+03	2.28E+04	9.62E+03	1.56E+04	9.58E+03	2.11E+04	4.73E+03	2.86E+04
f28	9.84E+03	5.55E+05	1.39E+04	9.37E+05	5.55E+04	7.26E+04	1.08E+04	9.41E+04	5.40E+03	9.77E+04
f29	1.09E+09	1.94E+10	2.93E+09	2.57E+10	7.24E+09	1.09E+10	1.20E+09	1.96E+10	1.53E+08	2.62E+10

**Table 3:** Statistical best ($\mu$) solutions of algorithms on the 29-CEC’2017 standard test suites.

F	SCA	Chimp	SBPO	AOA	IChoA
F1–23	$\mu$	$\mu$	$\mu$	$\mu$	$\mu$
f1	1.12E+11	1.94E+11	1.04E+11	5.89E+10	3.25E+10
f2	2.05E+04	2.46E+04	2.47E+04	2.40E+04	1.21E+04
f3	1.01E+05	1.85E+05	5.41E+04	3.88E+04	2.82E+04
f4	2.43E+03	2.66E+03	2.28E+03	2.03E+03	2.01E+03
f5	7.29E+02	7.37E+02	7.07E+02	7.03E+02	7.30E+02
f6	7.54E+03	9.52E+03	1.09E+04	3.88E+03	2.40E+03
f7	2.80E+03	3.06E+03	2.45E+03	2.38E+03	2.06E+03
f8	1.42E+05	1.88E+05	1.19E+05	8.55E+04	1.88E+04
f9	2.48E+03	3.13E+03	3.16E+03	2.82E+03	2.37E+03
f10	5.00E+05	2.45E+07	2.10E+05	2.09E+05	1.41E+05
f11	1.70E+11	2.45E+11	1.67E+11	8.77E+10	1.50E+10
f12	3.53E+10	6.62E+10	3.61E+10	1.39E+10	1.04E+10
f13	6.96E+07	2.01E+08	9.04E+07	5.01E+07	3.94E+07
f14	1.18E+10	2.70E+10	2.02E+10	6.21E+09	4.35E+09
f15	1.82E+04	3.34E+04	1.78E+04	1.52E+04	1.20E+04
f16	7.03E+06	1.70E+07	4.35E+06	2.65E+06	1.47E+06
f17	2.75E+08	3.14E+08	8.95E+08	2.25E+08	8.07E+07
f18	1.45E+10	2.35E+10	2.00E+10	7.79E+09	6.95E+09
f19	8.74E+03	9.21E+03	7.98E+03	8.79E+03	7.44E+03
f20	4.58E+03	4.87E+03	4.23E+03	4.09E+03	4.01E+03
f21	2.59E+03	4.26E+03	3.57E+03	2.92E+03	2.39E+03
f22	5.46E+03	6.33E+03	4.28E+03	6.81E+03	5.08E+03
f23	4.03E+03	4.50E+03	3.71E+03	4.35E+03	3.70E+03
f24	2.31E+04	4.31E+04	2.73E+04	9.20E+03	1.71E+03
f25	1.60E+04	2.19E+04	1.21E+04	1.42E+04	1.17E+04
f26	4.96E+03	5.94E+03	3.94E+03	4.78E+03	3.10E+03
f27	1.26E+04	1.63E+04	9.69E+03	9.93E+03	8.81E+03
f28	2.19E+04	2.31E+05	5.57E+04	1.27E+04	1.12E+04
f29	3.51E+09	1.06E+10	7.25E+09	1.63E+09	1.08E+09

**Table 4:** Statistical best (*sd*) solutions of algorithms on the 29-CEC’2017 standard test suites.

F	SCA	Chimp	SBPO	AOA	IChoA
F1–23	*sd*	*sd*	*sd*	*sd*	*sd*
f1	6.37E+10	1.18E+11	1.76E+09	2.18E+10	1.54E+09
f2	1.26E+05	2.09E+04	2.00E+04	2.35E+04	1.92E+04
f3	5.21E+04	1.14E+05	8.66E+03	1.73E+04	1.09E+04
f4	4.75E+02	4.78E+02	3.59E+01	9.39E+01	2.41E+01
f5	4.22E+01	3.06E+01	1.77E+00	6.88E+00	1.29E+00
f6	3.85E+03	4.53E+03	1.39E+02	6.49E+02	1.25E+02
f7	4.83E+02	5.72E+02	9.23E+00	1.11E+02	1.03E+02
f8	5.19E+04	8.05E+04	9.54E+02	1.06E+04	1.04E+02
f9	1.87E+02	1.21E+02	6.95E+00	1.67E+02	1.05E+02
f10	1.53E+06	9.04E+07	3.38E+03	4.71E+05	2.54E+03
f11	8.11E+10	1.21E+11	5.24E+09	3.04E+10	1.23E+09
f12	1.57E+10	3.45E+10	7.11E+09	8.29E+09	4.94E+09
f13	1.50E+08	7.40E+07	2.16E+07	3.92E+07	2.04E+07
f14	8.86E+09	1.45E+10	3.17E+09	3.68E+09	1.22E+09
f15	4.62E+03	1.68E+04	2.00E+02	1.84E+03	1.55E+03
f16	3.65E+07	2.41E+07	1.71E+06	1.93E+07	1.29E+06
f17	3.05E+08	2.76E+08	5.47E+08	2.09E+08	1.11E+08
f18	1.04E+10	1.12E+10	1.47E+09	5.34E+09	1.47E+09
f19	6.37E+02	8.17E+02	2.87E+01	4.51E+02	2.22E+01
f20	3.95E+02	3.74E+02	4.96E+01	1.87E+02	4.25E+01
f21	2.97E+02	2.64E+02	2.90E+02	1.75E+02	1.45E+02
f22	3.25E+02	8.36E+02	3.67E+01	7.04E+02	2.46E+01
f23	2.33E+02	4.91E+02	2.59E+01	2.63E+02	1.22E+01
f24	1.98E+04	2.77E+04	8.65E+02	3.13E+03	1.55E+02
f25	1.92E+03	7.10E+03	5.25E+02	1.35E+03	1.15E+03
f26	3.63E+02	1.09E+03	4.06E+01	8.14E+02	2.25E+01
f27	3.94E+03	6.15E+03	4.67E+02	1.58E+03	4.17E+02
f28	2.99E+04	3.76E+05	1.55E+03	1.00E+04	1.44E+03
f29	3.51E+09	8.36E+09	2.00E+08	2.12E+09	2.38E+08

#### Accuracy

In this subsection, we are discussing the statistical accuracy for the global optima score and performance of the proposed optimizer. The average value of each algorithm has been reported in Table 3. In Table 5, the average values have been illustrated into two different categories such as best (B) and worst (W) etc.These best and worst symbols have been assigned by the average values of Table 3. Normally the least average score shows the accuracy of the algorithm in finding the best optima in the search area. Results of Table 5, proof that the IChoA gives the least average value on maximum test suites as comparison than others. Hence it can be concluded that the IChOA method is able to provide the best or accurate solution for the complex suites than others.

**Table 5:** Statistical best ($\mu$) values of algorithms on the 29-CEC’2017 suites.

F	SCA	Chimp	SBPO	AOA	IChoA
f1	W	W	W	W	B
f2	W	W	W	W	B
f3	W	W	W	W	B
f4	W	W	W	W	B
f5	W	W	W	B	W
f6	W	W	W	W	B
f7	W	W	W	W	B
f8	W	W	W	W	B
f9	W	W	W	W	B
f10	W	W	W	W	B
f11	W	W	W	W	B
f12	W	W	W	W	B
f13	W	W	W	W	B
f14	W	W	W	W	B
f15	W	W	W	W	B
f16	W	W	W	W	B
f17	W	W	W	W	B
f18	W	W	W	W	B
f19	W	W	W	W	B
f20	W	W	W	W	B
f21	W	W	W	W	B
f22	W	W	B	W	W
f23	W	W	W	W	B
f24	W	W	W	W	B
f25	W	W	W	W	B
f26	W	W	W	W	B
f27	W	W	W	W	B
f28	W	W	W	W	B
f29	W	W	W	W	B

#### Stability

**Figure 2:**
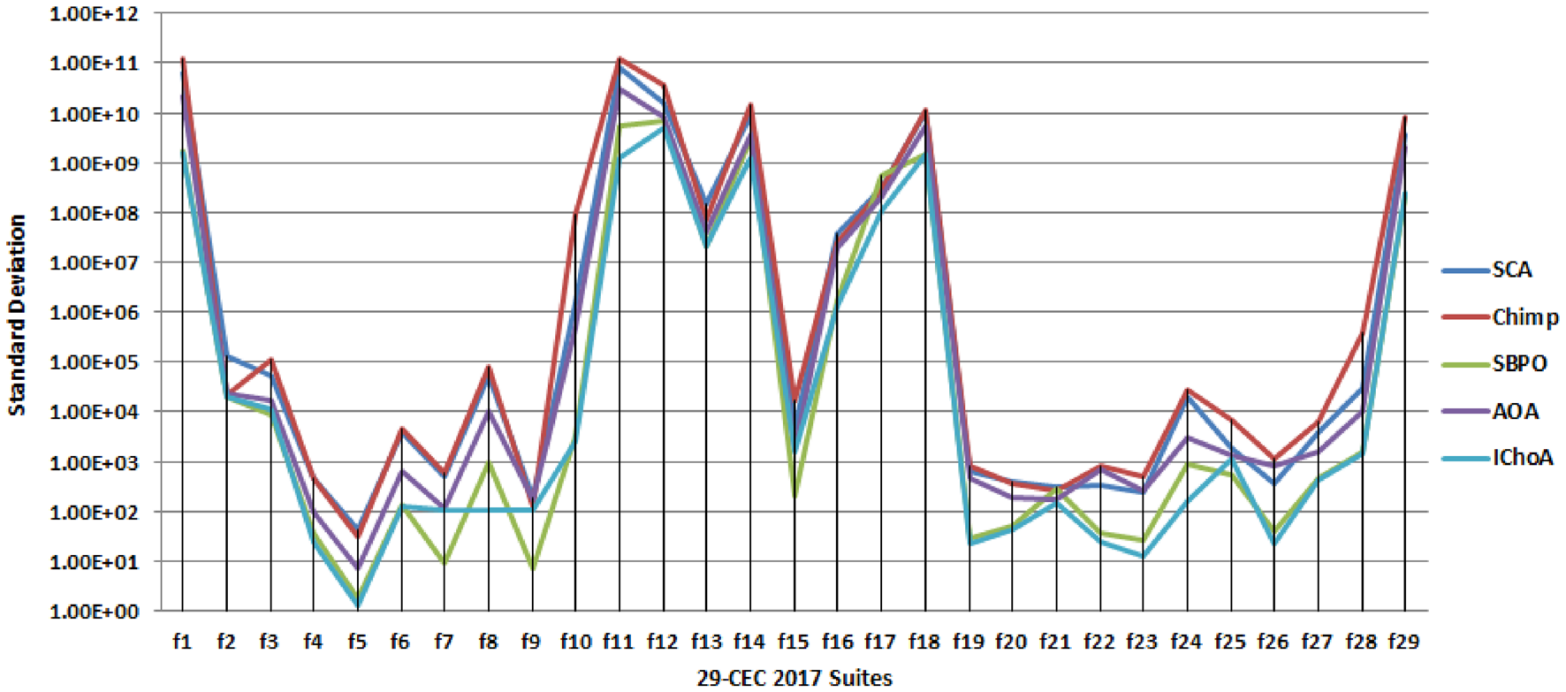
Statistical best (*sd*) solutions graph of algorithms on the 29-CEC’2017 suites.

In Fig. 2 the standard score of each method has been plotted. This graph has been plotted against the results of Table 4. If the standard score of algorithms lies near to zero this shows the stability of the algorithm for the optimal solutions of the given suites. In Table 4 and Fig. 2, it can be easily see that the IChoA is providing the least standard score at maximum test suites against the other methods. The least standard also shows the fast convergence speed of the algorithm for traps the optima in the search area. Hence the results of table and figure, gives the proof of the stability and fast convergence speed of the proposed method against others that the IChOA is able to trap the optima score fastly outperforms than others.

#### Convergence graphs analysis and discussion

**Figure 3:**
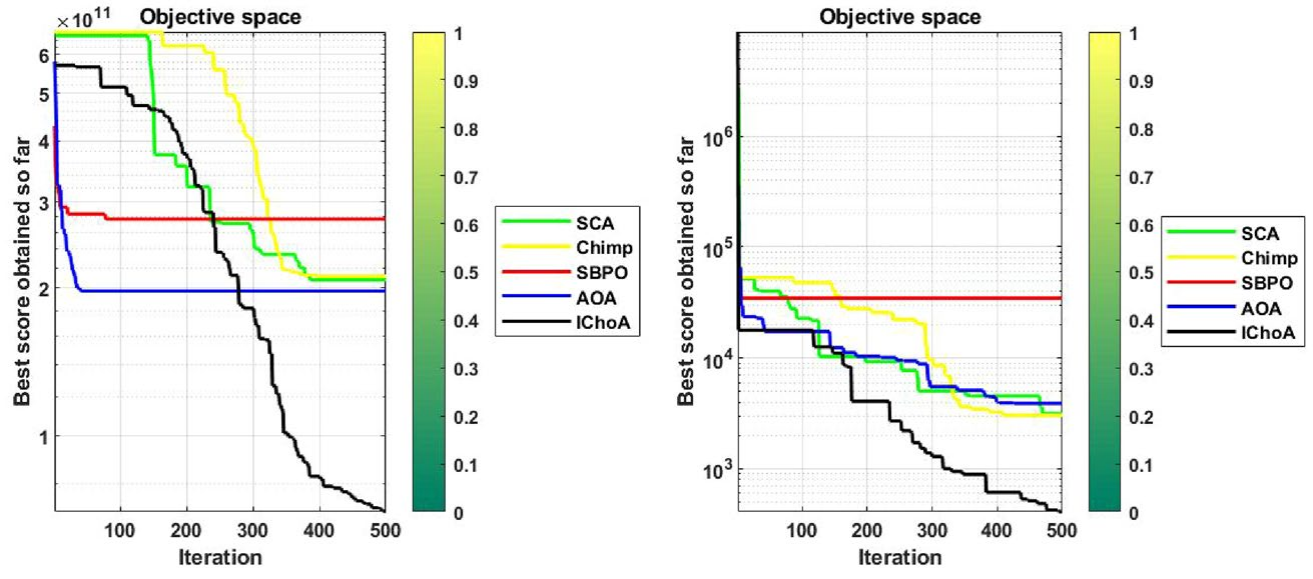
Convergence graphs of evolutionary algorithms on uni-modal test functions.

**Figure 4:**
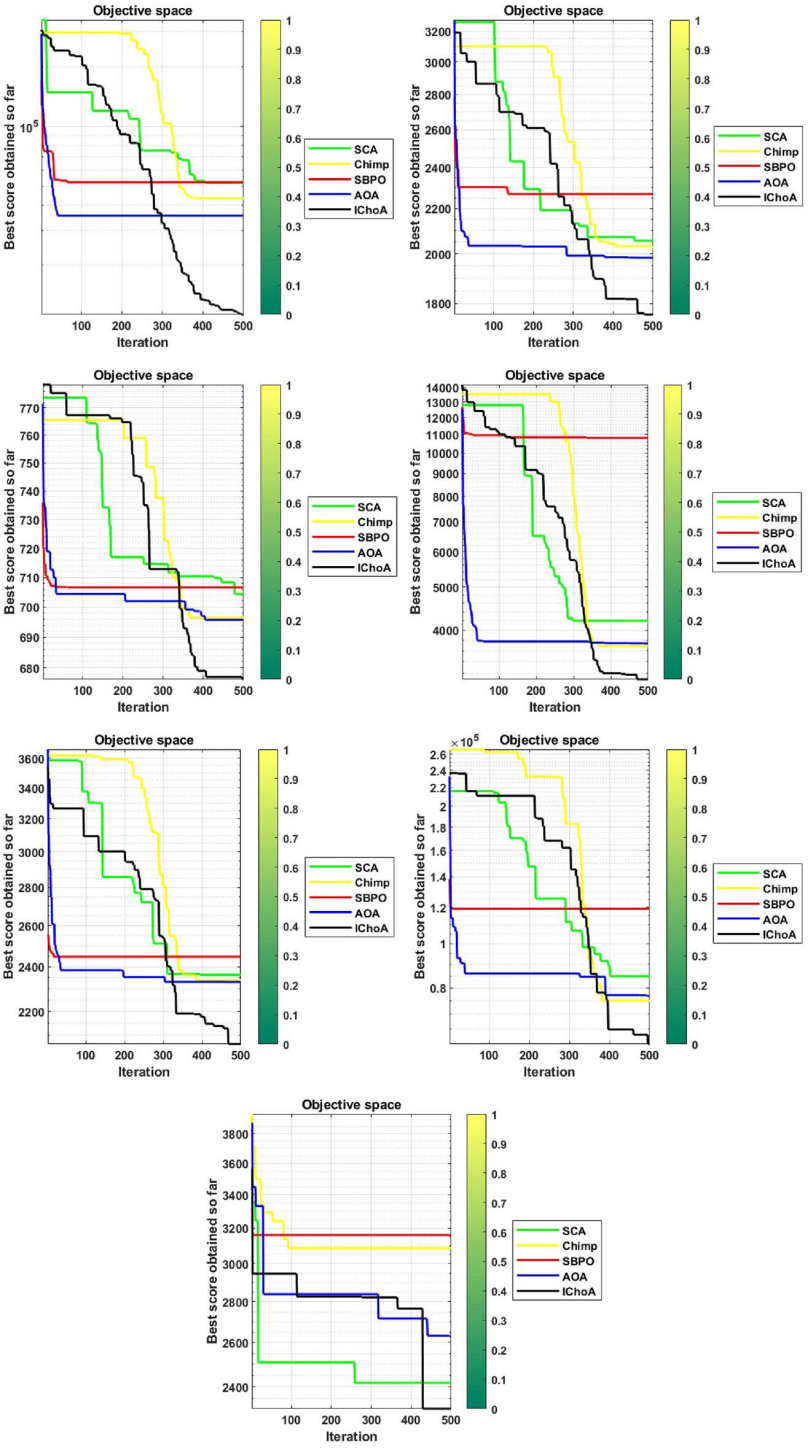
Convergence graphs of evolutionary algorithms on simple-multimodal test functions.

**Figure 5:**
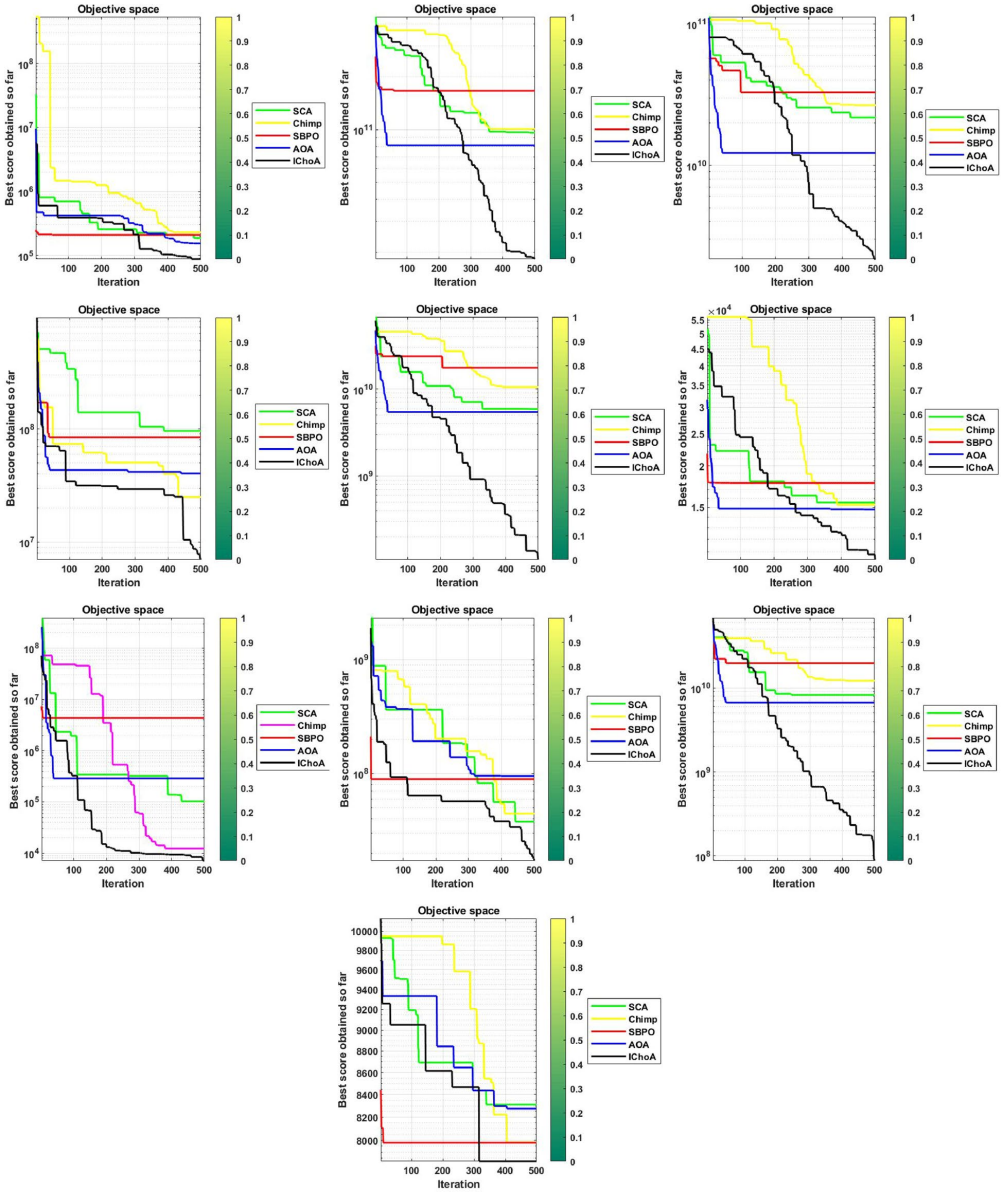
Convergence graphs of evolutionary algorithms on hybrid test functions.

**Figure 6:**
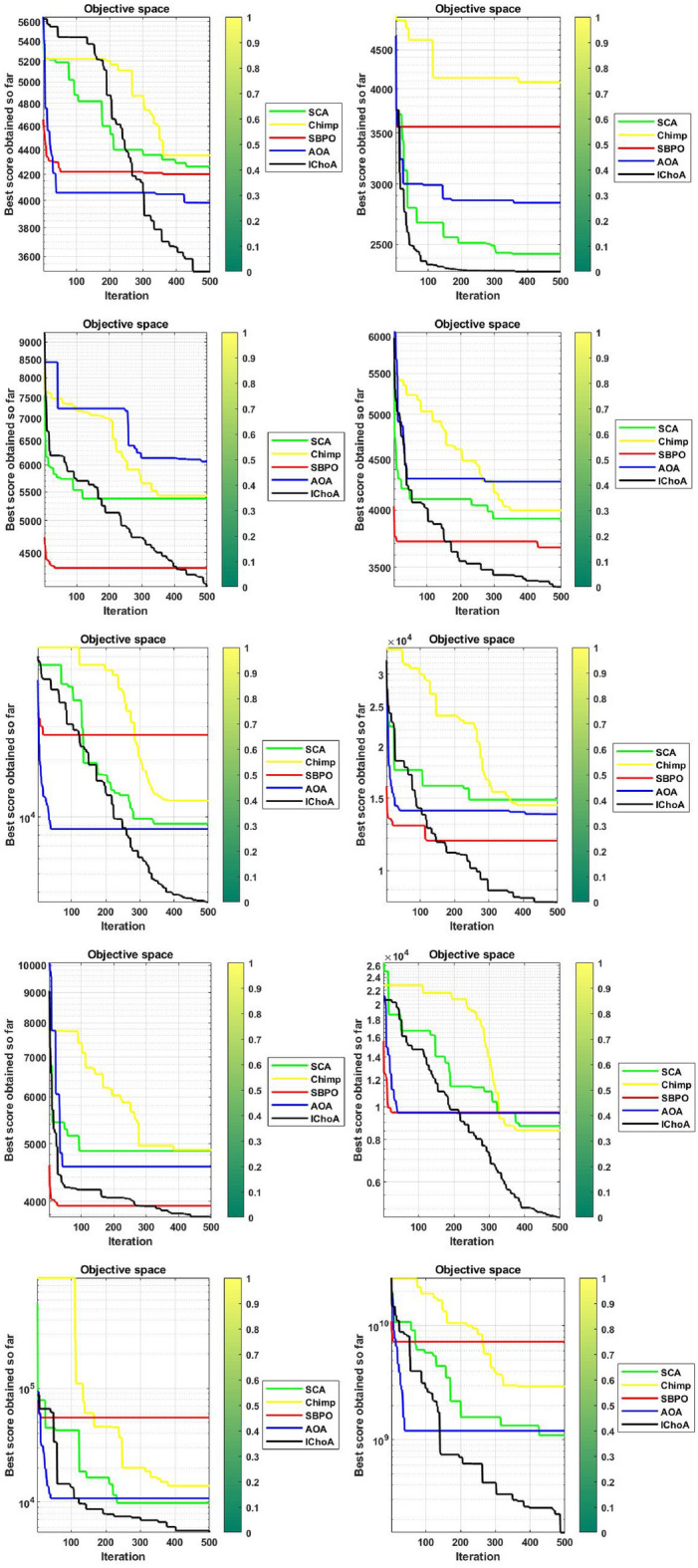
Convergence graphs of evolutionary algorithms on composition test functions.

The convergence performance graphs of algorithms are drawn against the number of the iterations and best solution so far etc and presented in Figs. 3, 4, 5, 6. Kindly keep in mind that the best optima that are obtained against every iteration which illustrated through the best solution so far. In Figs. 3, 4, 5, and 6, the x-axis denotes the number of iterations and y-axis illustrates the best solution found against each iteration during the search process.

As per V.D. Berg et al.^40^,this behaviour can guarantee that the evolutionary methods eventually touch to a target and find the local or global in a search domain. So, IChoA improves the fitness score for all chimps in a search area and assurance traps accurate targets for complex suites as iteration rises. Here can be said that this happens by this modification of the ChoA method. As each chimp travels from highest optima to lowest optima score, therefore with the hypothesis of growing in ChoA, the overall champs and their fitness values are amended against each iteration.

With this strategy we save the best score and apply these scores for finding the next best location for the entire swarm in the search area. Experiments and convergence graphs shows that the IChoA method is capable of giving the higher grantee finds the optima against the least number of iterations in a complex space as comparison to others. This behavior illustrates the fast convergence speed of the proposed method. So, the proposed strategy is to help the chimps for finding the better regions fastly of the complex suites.

## Parameter settings of energy constraints problem (ECP)

To design WSN, clustering module and the routing module is discussed here for the proposed network architecture. Proposed architecture utilizes dynamic clustering algorithm with hierarchical cluster formation to achieve the energy efficient routing. To start with the designing of the network, initial network parameters are created and listed in Table 6.

**Table 6:** Parameter settings for ECP.

Constant	Values
Size of network	100 m × 100 m
Number of nodes	100
Number of clusters	Variable
Initial energy of nodes	5 J
Location of nodes	Amid (0,0) & (100, 100)
Round time	20s
Simulation time	3600 s
Packet size	2000 bits
Maximum number of generations	500

## Formulation of energy constraints problem

To support the data aggregation in WSN, the network nodes can be accommodated in the small groups called the Clusters. Clustering can be defined as the division of the nodes in the groups on the basis of some mechanism. Clustering is done to achieve the energy efficiency and the scalability of the network. Formation of the cluster also involves the assigning the role to the node on the basis of their perimeters. Optimal clustering has a strong influence on the performance of WSN. Data aggregation is enabled at CH in order to discard the unwanted and uncorrelated data, thus saving the sensor node energy. As just CHs have to continue the local route set-up, routing can be managed easily and so just small routing information is required, this again enhances the scalability of the network to a significant extent. As the SNs communicate only with their CHs, communication bandwidth is also conserved thus avoiding the exchange of redundant messages within themselves.

The Energy constraint is a big challenging task in the field of WSNs. During these efforts we are trying to propose a new Low energy, routing and efficient scalable clustering protocol based on Metaheuristics in WSNs for improving the lifetime and enhancing the wireless sensor networks (WSNs) quality of service. In this proposed strategy has been taken new constants for optimizing the cluster heads selection. The proposed algorithm introduced various characteristics including energy, load on the node, weight of a node, scalability and balanced cluster respectively. Here the proposed method, gives the optimize path from the search agents of the cluster to the cluster head by selecting the most efficient path, going over sensors taking not as much of distance to attain data and extra energy. The brief details of these characteristics have been illustrated by the following mathematical equations.

The average energy of each sensor has been evaluated by the following equ. (26)^41^;

26 $$\begin{aligned} E_{A}= \frac{e_{s_{1}}+e_{s_{2}},\ldots ,e_{s_{n}}}{n}=\frac{\sum _{i=1}^{n}e_{s_{i}} }{n} \end{aligned}$$

where *n* and $e_{s_{i}}$ illustrates the number and energy of the node.

We find the all nodes nearby the neighborhood or locality of the node $S_{i}$. After that we are evaluated the distance separating these nodes and $S_{i}$.At this stage a afresh constant is introduce such as weight (*W*).This parameter and constant is calculated by the following equ.(27)^41^;

27 $$\begin{aligned} W_{S_{i}}=\frac{E_{S_{i}}}{\sum _{d=n}^{m}d(S_{i},S_{n})+\cdots +d(S_{i},S_{n})} \end{aligned}$$

where *d* is denotes the distance of the nodes and $E_{S_{i}}$ and its neighbors respectively.

For creating the relationship amid the living and neighborhood nodes the following Eq. (28) has been applied^41^;

28 $$\begin{aligned} S_{s_{i}}=\frac{N_{S_{i}}}{n} \end{aligned}$$

where $N_{S_{i}}$ indicates the no’s of neighbors of the node *S* and *n* denotes the no’s of living nodes. After calculating the node’s $S_{s_{i}}$ the following leach Eq. (29) has been applied for verifying the solution^41^;

29 $$\begin{aligned} L(n)=\left\{ \begin{array}{ll} \frac{p}{1-p\left\{ r\,\,mod(\frac{1}{p}) \right\} } &{} n\epsilon M\\ 0 &{} n\epsilon M \end{array} \right. \end{aligned}$$

where *p* denotes the probability of cluster head in all round which is the ratio of the nodes and no’s of cluster head. Further *r* and *M* are illustrated the recent round number and set of the nodes.

The lode of routed packets have been calculated by the following equation;

30 $$\begin{aligned} L_{i}=\frac{\sum _{k=0}^{w}R_{pk}}{time} \end{aligned}$$

where $L_{i}$ illustrates the load of *i*-th routed packets and $R_{pk}$ denotes the routed packets respectively.

The fitness function has been illustrated by the Eq. (31).This function is developed by the above equations. On the basis of this function we are calculating the maximum weight and neighboring life of the node sensor $S_{i}$ etc.

31 $$\begin{aligned} F_{1}=Max(W)+Max(S_{s_{i}}) \end{aligned}$$

On the basis of load function (30) has been derived the following new function for calculating the maximum load on the nodes;

32 $$\begin{aligned} f(c,i)= & {} \sum _{i=1}^{n}\frac{E_{c,i}h_{c,i}\times max\left( \sum _{k=0}^{w} w_{c,i}\right) }{L_{i}} \end{aligned}$$

33 $$\begin{aligned} F_{2}= & {} Min(L_{i})+Max( f(c,i)) \end{aligned}$$

where $E_{c,i}$ and $h_{c,i}$ are denotes the energy and health of the nodes.

For intra-cluster distance has been applied the following Eq. (34). To most effective and well-organized networks converge this function must be needed to minimized. Since the least intra-cluster distance (ICD) shows the energy spent of each cluster for the communication of files or data^42^.

34 $$\begin{aligned} F_{3}=\sum _{k=1}^{K}cd_{N}^{k} \end{aligned}$$

where ICD for *k*th cluster with *N* no’s of sensor networks is calculated by the following equation;

35 $$\begin{aligned} cd_{N}^{k}=\sum _{i=1}^{N}d_{s_{i}}^{ch_{k}} \end{aligned}$$

Here, this function have been applied for evaluating the total distance between sensor networks and cluster heads in the network.

The scalability of the network has been evaluated through the following Eq. (36).This formulation shows the capability of the protocol to deliver the constant message facilities while density and size of network differs^42^.

36 $$\begin{aligned} S=\sum _{i=1}^{K}\sum _{j=1}^{K}d_{ch_{i}}^{ch_{i}} \end{aligned}$$

During this work if objective function is applied for minimize problem then the average ICD can be defined by the following Eq. (37)^42^;

37 $$\begin{aligned} F_{4}= \frac{1}{S} \end{aligned}$$

The least distance between ICD and each cluster head shows the balance of the clustering. For this purpose the following mathematical formulation has been applied^42^;

38 $$\begin{aligned} F_{5}= \sqrt{\frac{\sum _{k=1}^{K}\left( cd_{k}-\bar{cd} \right) ^{2}}{K-1}} \end{aligned}$$

where $cd_{k}$ denotes the average ICD of the network and $\bar{cd}$ illustrates the ID of *k*th cluster.

Finally, for the objective of achieving the energy well-organized network, balanced cluster and scalability development the following fitness function has been applied^42^.

39 $$\begin{aligned} Min(F)=F_{6}=\mu _{1}\times F_{3}+\mu _{2}\times F_{4}+\mu _{3}\times F_{5} \end{aligned}$$

where $\mu _{1}$,$\mu _{1}$ and $\mu _{3}$ are denoted the weightage constants as $\mu _{1}+\mu _{2}+\mu _{3}=1$.

## Implementation

During this work, the proposed method has been implemented on the $F_{1} - F_{5}$ functions to achieving the maximum weight, neighboring life of the node sensor, maximum load on the nodes, the energy well-organized network, balanced clustering and scalability develop respectively. The implementation steps of IChoA based cluster head selection has been illustrated through Algorithm 3;

### Pseudocode of IChoA based cluster head selection algorithm

The pseudocode of Improved Chimp Optimizer is reported in Algorithm 3.
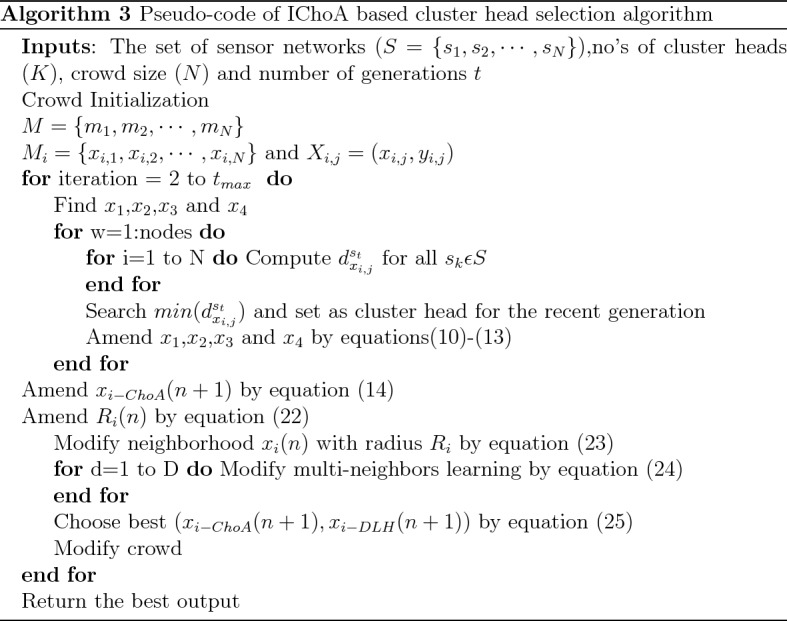


## Simulation outputs and discussion

The routine of the clustering protocol is depending on the objective functions. The main objective of this work is to produce a good cluster in a least network area and poor clusters in a large network area. For obtaining this objective here we are implementing a various population based method such as GA^43^, ALO^44^, OBCA, Chimp^35^, SCA^36^.

The performance of the energy efficient scalable clustering protocol (EESCP) in WSNs has been tested and verified by the various methods such as GA, OBCA, ALO, Chimp, SCA and IChoA respectively. The robustness of the algorithms have been verified in the terms of energy consumption, balanced clustering, network life, throughput, best least and highest fitness values etc. In the following subsections the brief explanation has been presented on the output solutions of the algorithms.

**Figure 7:**
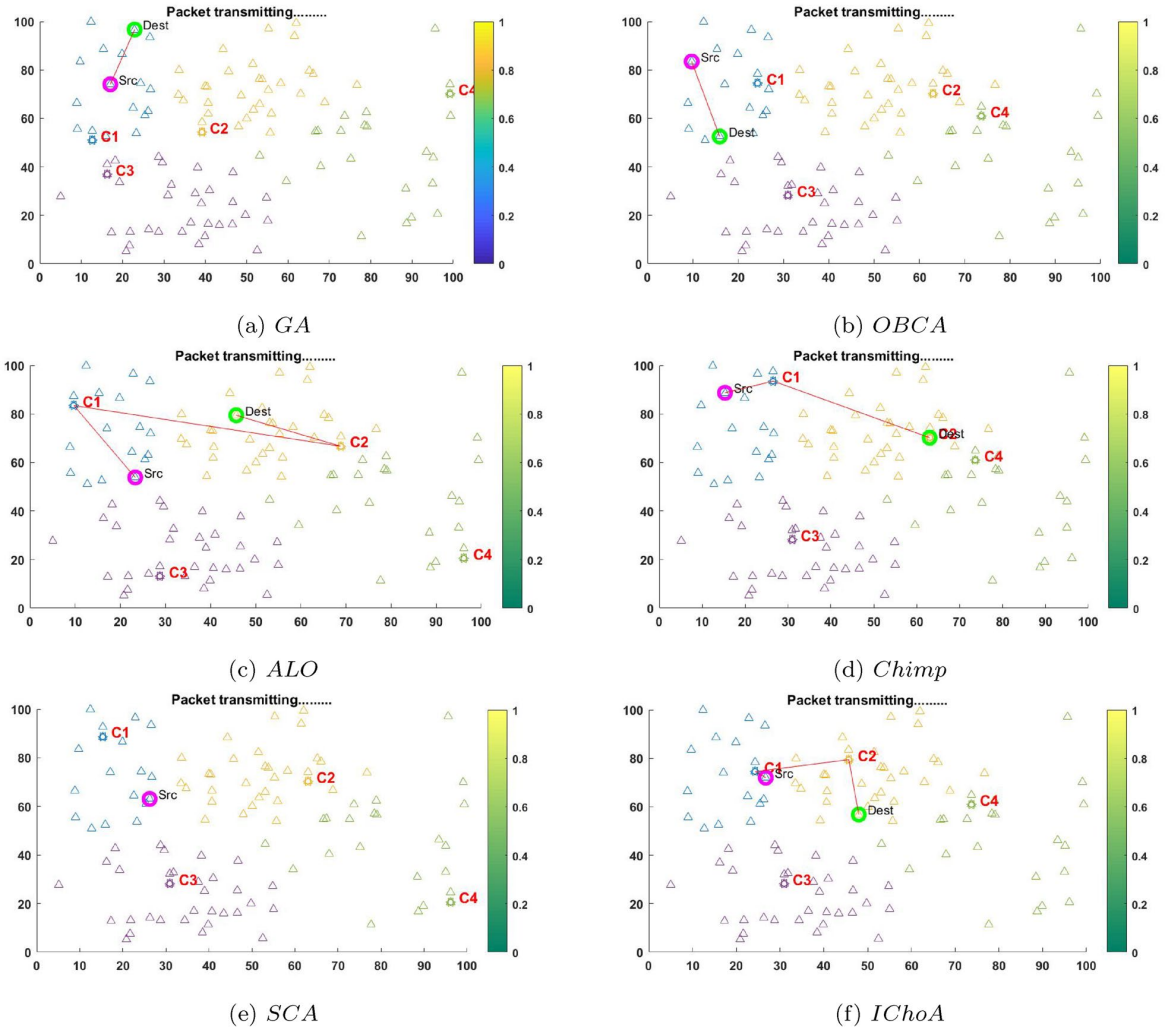
The packet transmitting graphs of metaheuristics on $F_{2}$.

**Figure 8:**
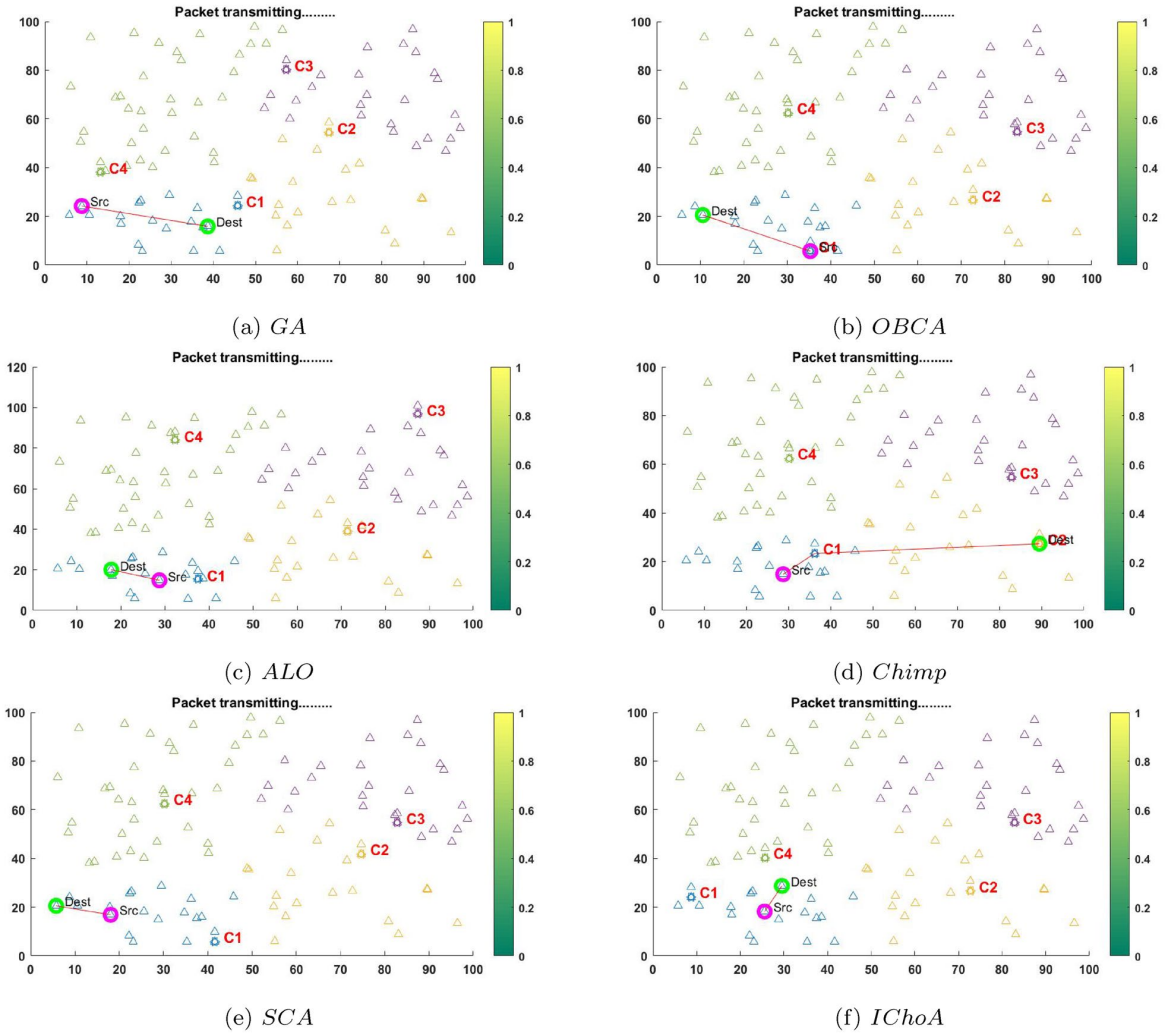
The packet transmitting graphs of metaheuristics on $F_{6}$.

The communication plot for packet transmission is shown in Figs. 7 and 8 using $F_{2}$ and $F_{6}$ for randomly deployed sensor nodes and cluster heads.The sensor nodes for proposed network architecture are deployed with the initial parameters. The robustness of the algorithms has been verified in the terms of packet loss, energy consumption, balanced clustering, network lifetime, throughput, best least and highest fitness values etc. Figures 7 and 8 shows the plotting of various nodes and cluster heads. The CH from each cluster can be selected randomly with every iteration and is chosen particularly on runtime. The CH selection is done dynamically and performance is optimized using IChoA on the basis of residue energy on random basis. Also, Figs. 7 and 8 illustrate the plotting of the CH selection is done dynamically and performance is optimized using GA, ALO, SCA, OBCA and Chimp optimization metaheuristics on the basis of available node energy. Here the cluster head among each cluster can be selected in every iteration on the basis of residual energy and distance between the nodes. The coverage is calculated by using distance vector that routes packets in efficient manner. The sensed data by the sensors is transmitted between the source and the destination over the routes formed by coverage vector. All the routes are calculated by the shortest path selection modules with graph theory. The performance of the proposed energy efficient clustering protocol using IChoA in WSNs has been tested and verified by the various methods such as GA, OBCA, ALO, Chimp, SCA and IChoA respectively.

### Throughput of network

**Figure 9:**
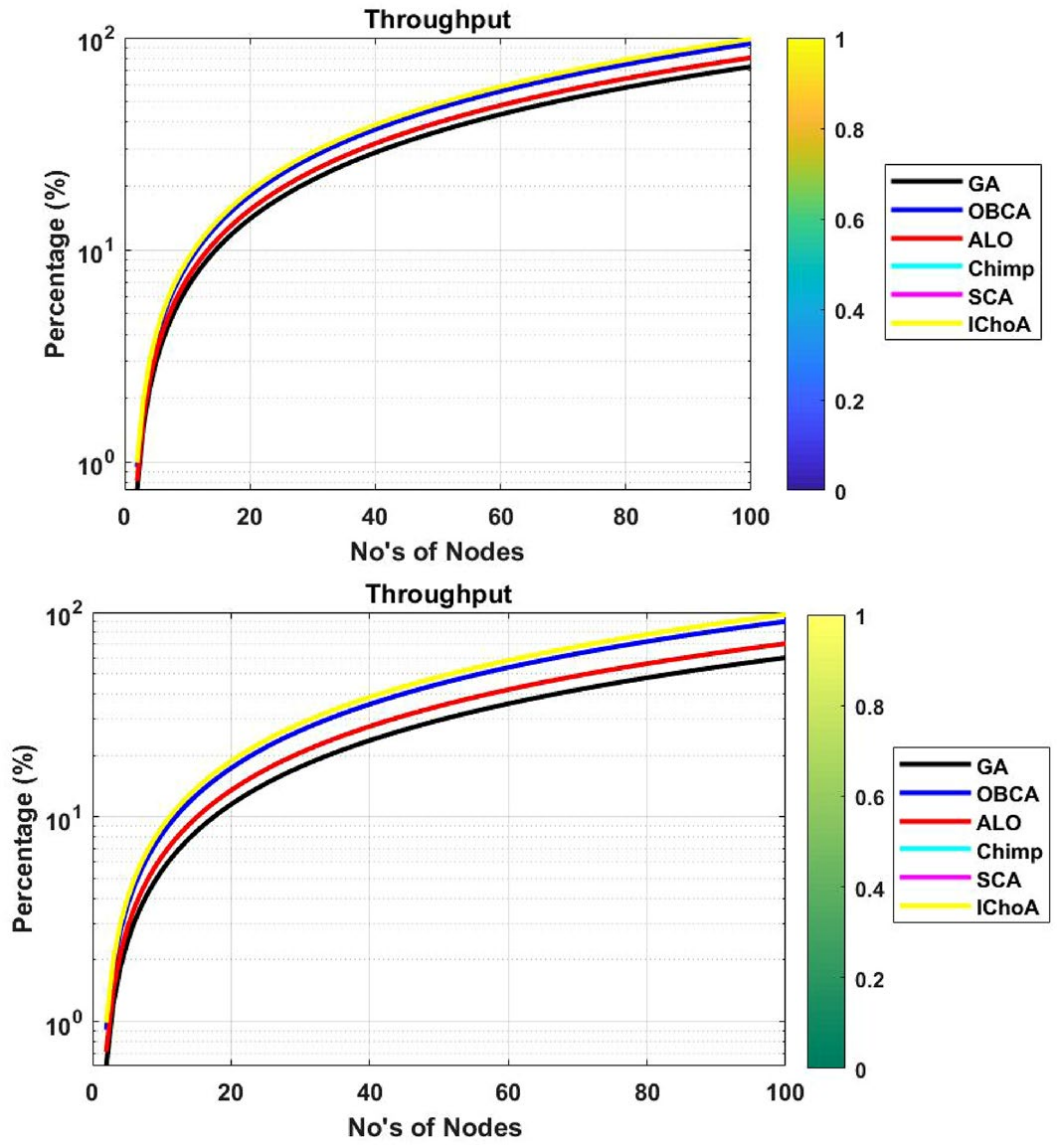
Throughput graphs of metaheuristics on functions $F_{2}$ and $F_{6}$.

Here the throughput of the network is verifying the total number of packets acknowledged at base station in its period. The solutions of the algorithms are reported in Table 7 and graphs illustrated by Fig. 9. In Table 7, we can easily see that the proposed method is able to provide the 98.6261 at $F_{2}$ and 97.7061 at$F_{6}$ maximum number of packets at base section in its period or lifetime as comparison to others. So it could be concluded that EESCP obtains more packets by proposed method than other clustering protocols.

**Table 7:** Throughput outputs of metaheuristics on $F_{2}$ and $F_{6}$.

Method	$f_{min}$	$f_{max}$	$\mu$	$\sigma$
**F**$_{2}$				
GA	0	73.0485	36.5242	21.4065
ALO	0	80.5492	40.2746	23.6046
OBCA	0	93.8689	46.9344	27.5078
Chimp	− 76.8954	0	− 38.4477	22.5339
SCA	− 325.3948	0	− 162.6974	95.3554
IChoA	0	98.6261	29.3131	20.9019
**F**$_{6}$				
GA	0	60.0199	30.01	17.5886
ALO	0	70.331	35.1655	20.6102
OBCA	0	90.2154	45.1077	26.4372
Chimp	− 61.0316	0	− 30.5158	17.885
SCA	0	38.9023	33.9056	29.135
IChoA	0	97.7061	28.853	16.6323

### Average time consumption

**Figure 10:**
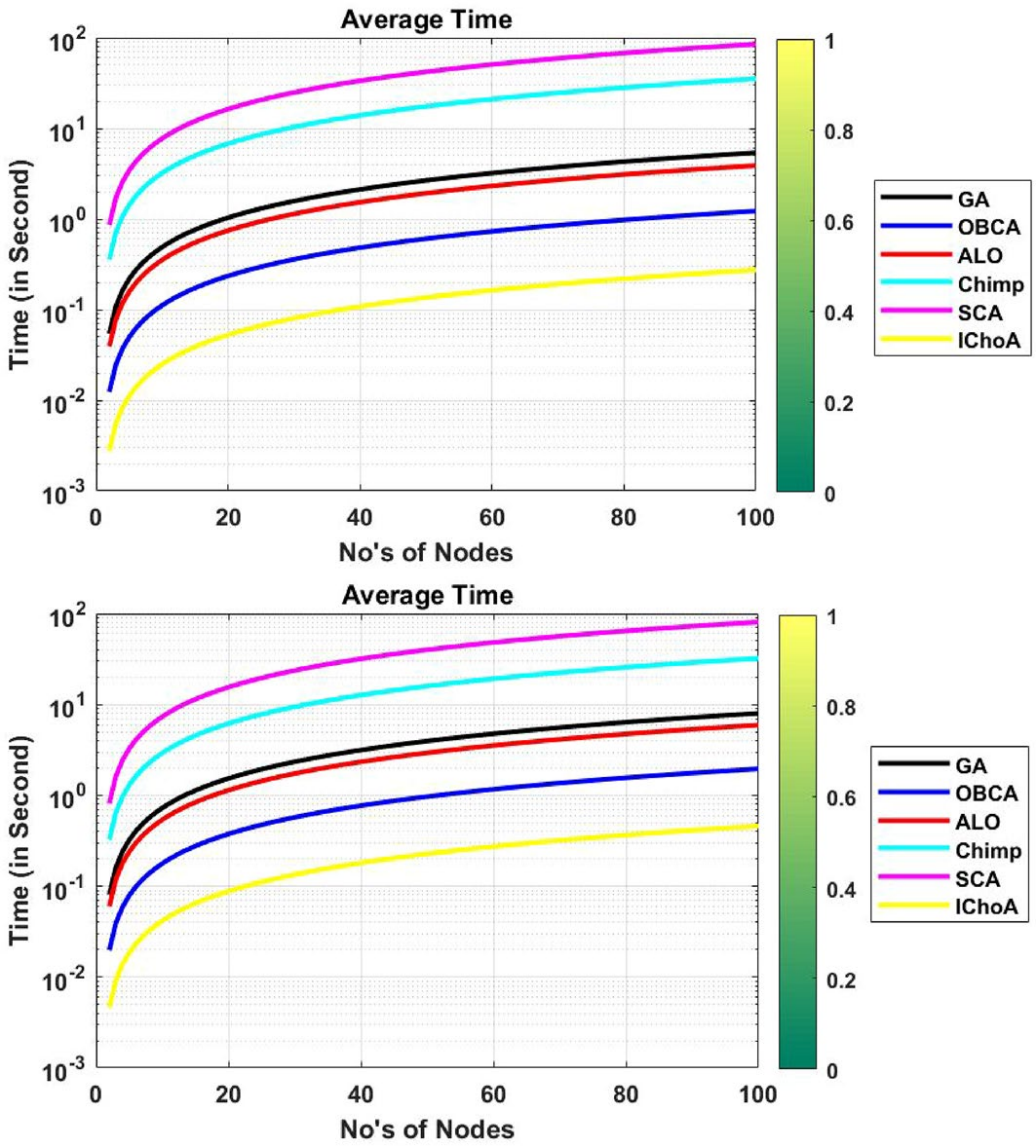
Average time consumption graphs of metaheuristics on functions $F_{2}$ and $F_{6}$.

In this subsection, we are comparing the total time consumption of the algorithms between all the sensor networks and cluster heads of the network. Experimental results are illustrated in Table 8. And the average time consumption for the data transformation by all algorithms has been plotted by Fig. 10. In this Table 8 and Fig. 10, it can be easily seen that the proposed method has taken least time to transfer the data of the network per round as compared to other methods. So it can be concluded that the IChoA method is able to transfer the data of the network fastly

**Table 8:** Average time consumption outputs of metaheuristics on $F_{2}$ and $F_{6}$.

Method	$f_{min}$	$f_{max}$	$\mu$	$\sigma$
**F**$_{2}$				
GA	0	3.8902	1.9451	1.14
ALO	0	4.7689	3.5689	6.4532
OBCA	0	1.2262	0.6131	0.3593
Chimp	0	35.3791	17.6895	10.3677
SCA	0	85.079	42.5395	24.932
IChoA	0	0.2748	0.1374	0.0805
**F**$_{6}$				
GA	0	4.6789	1.7654	1.8976
ALO	0	5.9338	2.9669	1.7389
OBCA	0	1.9569	0.9785	0.5735
Chimp	0	32.2063	16.1032	9.4379
SCA	0	1.9569	0.9785	0.5735
IChoA	0	0.4588	0.2294	0.1344

as compared to others. And with the help of this strategy we can transfer the data of the network per round fastly.

### Network lifetime

**Figure 11:**
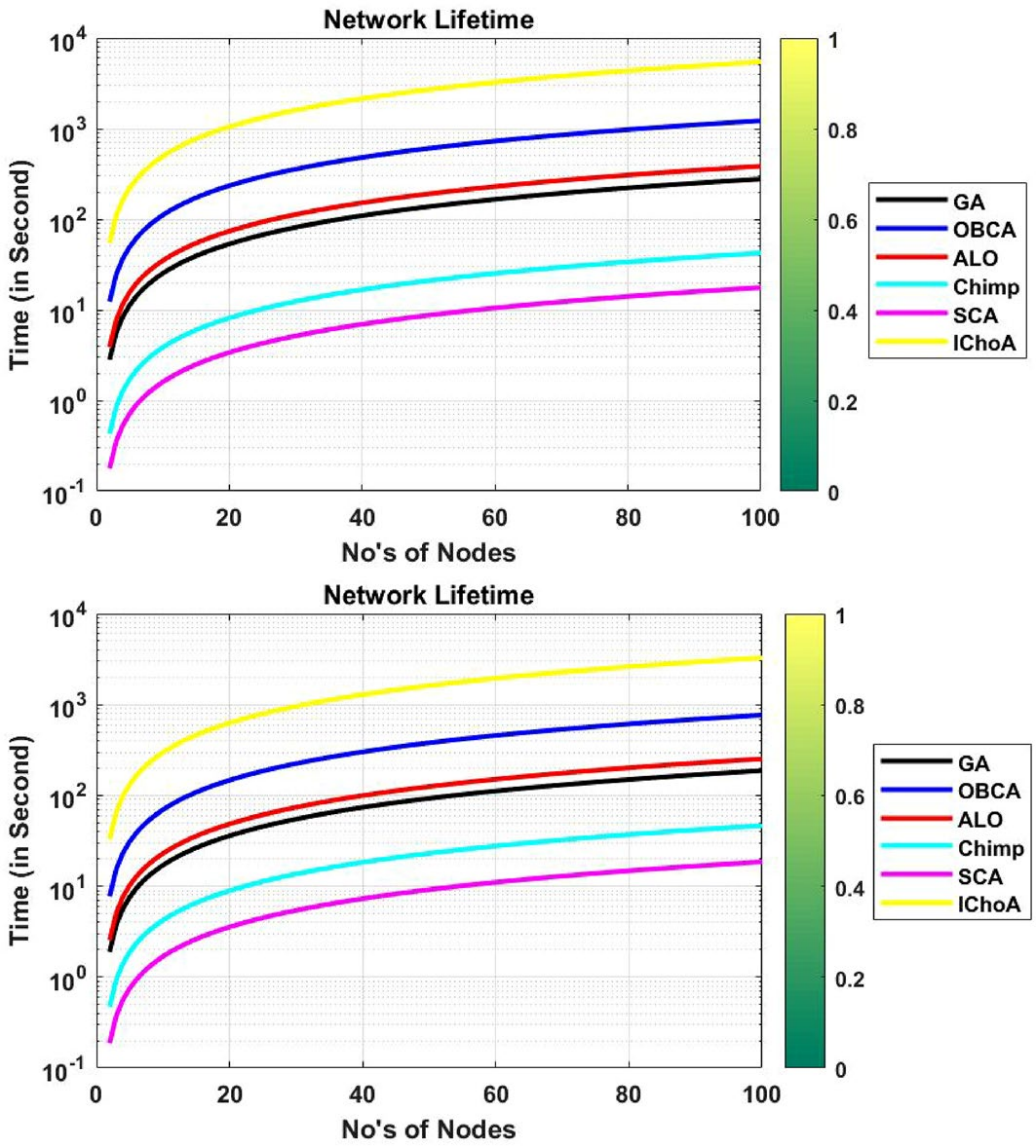
Network life graphs of metaheuristics on functions $F_{2}$ and $F_{6}$.

In the literature various methods are applied for measuring the lifetime of a network. Under this work we are applying the various recent robust optimizers for evaluating the lifetime of the network. Numerical results of all the algorithms on $F_{2}$ and $F_{6}$ has been illustrated in Table 9. The lifetime graph of a network is reported by Fig. 11. The results and graphs show that the IChoA method gives the highest lifetime of the network as compared to others. So this strategy could help in increasing the lifetime of the network.

**Table 9:** Network life outputs of metaheuristics on $F_{2}$ and $F_{6}$.

Method	$f_{min}$	$f_{max}$	$\mu$	$\sigma$
**F**$_{2}$				
GA	0	278.2773	139.1386	81.5479
ALO	0	385.5887	192.7943	112.995
OBCA	0	1.22E+03	611.6326	358.4722
Chimp	0	42.3979	21.199	12.4245
SCA	0	17.6307	8.8153	5.1666
IChoA	0	5.46e+03	2.73e+03	1.60e+03
**F**$_{6}$				
GA	0	187.5935	93.7968	54.9734
ALO	0	252.7888	126.3944	74.0786
OBCA	0	7.67E+02	383.2537	224.6214
Chimp	0	46.5747	23.2874	13.6485
SCA	0	18.5566	9.2783	5.4379
IChoA	0	3.27e+03	1.63e+03	9.58e+02

### Packet loss

**Figure 12:**
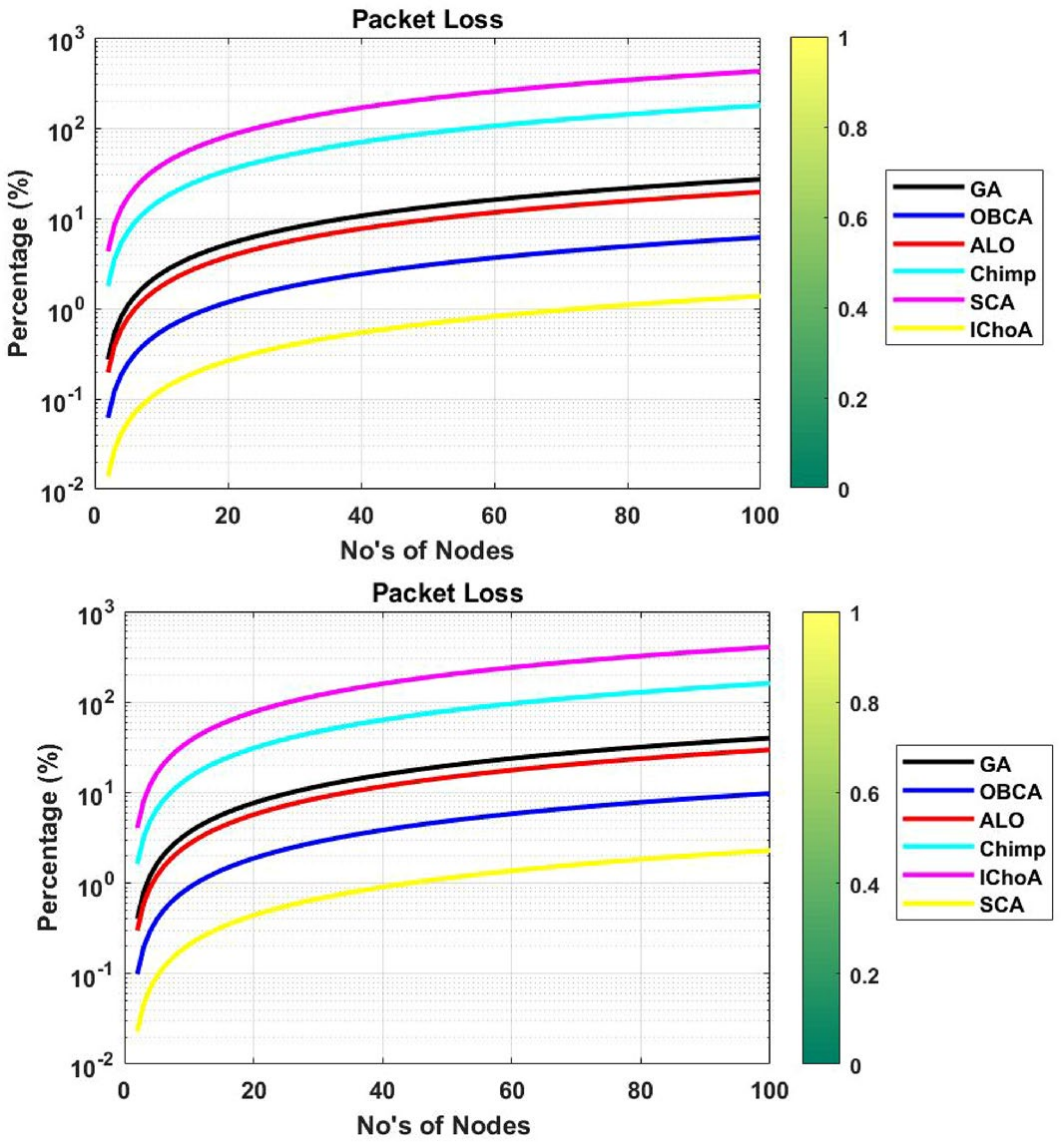
Packet loss graphs of metaheuristics on functions $F_{2}$ and $F_{6}$.

In this subsection, we are measuring the total packet loss during the transfer of data of one source to another source. The results of the table shows that the IChoA is able to transfer the highest data with the least number of packet loss. Experimental results and graphs of algorithms in Table 10 and Fig. 12 shows that the IChoA total packet loss is very less than other methods. Hence it can be concluded that the proposed method is able to give the strong network with least packet loss.

**Table 10:** Total packet loss outputs of metaheuristics on $F_{2}$ and $F_{6}$.

Method	$f_{min}$	$f_{max}$	$\mu$	$\sigma$
**F**$_{2}$				
GA	0	26.9515	13.4758	7.898
ALO	0	19.4508	9.7254	5.7
OBCA	0	6.1311	3.0656	1.7967
Chimp	0	176.8954	88.4477	51.8384
SCA	0	25.3948	15.6974	124.66
IChoA	0	1.3739	0.6869	0.4026
**F**$_{6}$				
GA	0	39.9801	19.99	11.716
ALO	0	29.669	14.8345	8.6944
OBCA	0	9.7846	4.8923	2.8673
Chimp	0	161.0316	80.5158	47.1896
SCA	0	404.168	202.084	118.4396
IChoA	0	2.2939	1.147	0.6722

### Energy consumption

**Figure 13:**
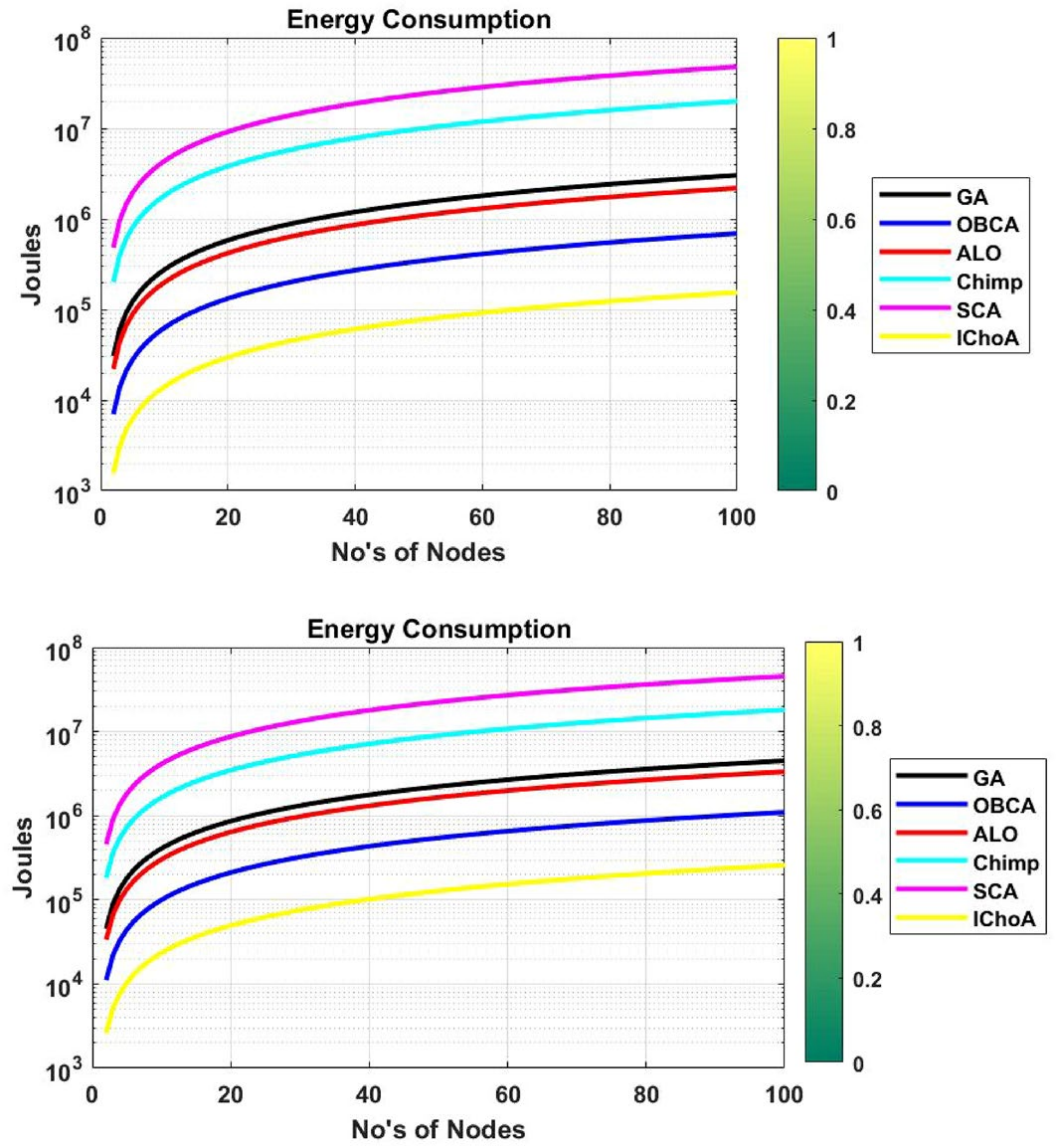
Energy consumption graphs of metaheuristics on functions $F_{2}$ and $F_{6}$.

In this phase, we are measuring the energy consumption of algorithms by all the sensor networks and cluster heads of the network per round. The results of the algorithms are reported in Table 11. And the energy consumption graphs of the algorithms are plotted by Fig. 13. Experimental results and graphs show that the proposed method is consuming the least energy during transfer of the data from one source to another. However, the other methods consume huge energy as compared to the proposed method. So on the basis of the result of Table 11 and Fig. 13 it could be concluded that the proposed method is competent in transferring the data with a least consumption of energy.

**Table 11:** Total energy consumption outputs of metaheuristics on $F_{2}$ and $F_{6}$.

Method	$f_{min}$	$f_{max}$	$\mu$	$\sigma$
**F**$_{2}$				
GA	0	3.03E+06	1.52E+06	8.89E+05
ALO	0	2.19E+06	1.09E+06	6.41E+05
OBCA	0	6.90E+05	3.45E+05	2.02E+05
Chimp	0	1.99E+07	9.95E+06	5.83E+06
SCA	0	4.79E+07	2.39E+07	1.40E+07
IChoA	0	1.55E+05	7.73E+04	4.53E+04
**F**$_{6}$				
GA	0	4.50e+06	2.25e+06	1.32e+06
ALO	0	3.34e+06	1.67e+06	9.78e+05
OBCA	0	1.10e+06	5.51e+05	3.23e+05
Chimp	0	1.81e+07	9.06e+06	5.31e+06
SCA	0	4.55e+07	2.27e+07	1.33e+07
IChoA	0	2.58e+05	1.29e+05	7.56e+04

### Accuracy of outputs

Generally the least mean $\mu$ score represents the accuracy of the solutions which are obtained by the algorithms. Results of Tables 7, 8, 9, 10 and 11 give strong evidence that the proposed algorithm is finding the solutions with least average score as comparison than others. So, it could be concluded that the IChoA method is capable of providing the best and accurate solution of the issue of energy constraint.

### Stability

The least standard score presents to the convergence speed and stability of the algorithms. Results of tables, shows that the proposed method is finding the best destination for energy constraint issues against the least standard score. Hence on the basis of obtained solutions we can conclude that the IChoA algorithm traps the destination fastly with at least time as comparison to others. Additionally, the least standard scores prove the stability of the proposed method for energy constraint issues.

### Performance evaluation of IChoA with various existing algorithms

The simulated performance evaluation of the proposed IChoA algorithm for computing analysis of various parameters such as network lifetime, average execution time, packet loss energy consumption and throughput are provided by Table 12.

**Table 12:** Performance evaluation of various optimization algorithms simulated for 100 nodes wireless sensor network.

Units	(s)	(%)	(pkt %)	(s)	(J)
Algorithms	Execution time	Throughput	Packet loss	Network life	Energy
ALO	6.5	17.05	32.96	227.62	3.7
GA	8.75	32.22	43.77	171.34	4.9
Chimp	34.56	26.88	172.82	43.39	1.95
SCA	101.7	14.44	508.55	24.98	5
OBCA	4.2	49.47	12.78	712.27	1.18
Proposed IChoA	2.12	99.8	15	3268.13	0.2

The performance in percentage is listed in Table 13 for various optimization algorithms in WSN. The result revealed that network lifetime of the proposed IChoA is improved 89% times as compared to existing optimization algorithms GA (18%), ALO (14%), OBCA (62%), Chimp (11%) and SCA (1%).

**Table 13:** Performance evaluation of various optimization algorithms for wireless sensor network (%).

Algorithms	Execution time (%)	Throughput (%)	Packet loss (%)	Network life (%)	Energy (%)
ALO	9	17	10	14	34
GA	6	32	5	18	26
Chimp	24	27	24	11	23
SCA	64	14	64	1	24
OBCA	3	49	2	62	7
Proposed IChoA	1	99	1	89	1

The proposed IChoA is using CH is selected with the less execution time only of the order of 1% in comparison to the other existing optimization algorithms GA (6%), ALO (9%), OBCA (3%), Chimp (41%) and SCA (64%). Also, the proposed IChoA algorithm consumes less time as compared with other approaches that leads to increase in the network speed which in turn decreases the average computation time. The proposed IChoA provides only 1% times the packet loss that is appreciable for the network data aggregation as compared to existing optimization algorithms GA (5%), ALO (10%), OBCA (2%), Chimp (24%) and SCA (64%). The energy consumption for the proposed IChoA is very less as compared to the other simulated methods and enhances the longevity of the network by consuming only 1% of the energy as compared to the other existing optimization algorithms GA (26%), ALO (34%), OBCA (7%), Chimp (23%) and SCA (24%). The throughput for the proposed IChoA is very high as compared to the other simulated methods and the packets reach the destination node successfully with minimum delay to increase capacity of the network by providing only 89% of the throughput as compared to the other existing optimization algorithms GA (14%), ALO (17%), OBCA (69%), Chimp (17%) and SCA (10%).

Summing up, all the efforts show that the IChoA method is able to tackle complex real suites and energy constraint issues. Hence, this strategy could be helpful for resolving the complex issues related to different domains of the different fields.

## Conclusion

WSNs that proliferate various real world applications which can be serious such as surveillance of military or army battle field, health-care and face the task of restricted energy capacity. To resolve the issues of the energy constraint based on chimp optimizer, namely improved chimp optimizer (IChoA) is developed. This strategy has been introduced by integration of Chimp Optimizer and dimension learning based hunting (DLH). The DLH phase helps the ChoA algorithm for maintaining diversity and also improves the balance between exploitation and exploration.To evaluate the effectiveness of the proposed method, it has been tested on 29-CEC-2017 and issues of energy constraints. Accepting the accurate choice of modification, fitness functions and IChoA operations have controlled longer system operability and extreme data rate with least difficulty. All simulations prove that the robustness and performance of the IChOA is superior to other recent methods in terms of minimum and maximum objective function values, mean, average, energy consumption, loss packets, average time, throughput and network lifetime respectively.

In future direction, the enhanced and hybrid optimizers will be introduced for complex energy constraints and engineering applications.

## Data Availability

The data that support the findings of this study are available from the corresponding author upon reasonable request.

## Abbreviations

$$\mu$$: Mean
sd & $\sigma$: Standard deviation
B: Best value
W: Worst value
s: Seconds
J: Jouls
$$w_{1}$$, $$w_{2}$$ & $$w_{3}$$: Weightage constants
$f_{min}$: Minimize objective function value
$f_{max}$: Maximize objective function value
$L_{i}$: Load on *i*th node
$$E_{A}$$: Average Energy
m: Choatic vector
$$E_{c}$$: Residual Energy
$h_{c}$: Health of the node
$$W_{c}$$: Coverage Weight
ICD: Intra Cluster distance
k: Cluster
N: Number of nodes
